# Designing novel linagliptin analogs through combined generative artificial intelligence, molecular docking, molecular dynamics simulation, and binding energy estimation for targeting fibroblast activation protein

**DOI:** 10.3389/fchem.2026.1848970

**Published:** 2026-09-09

**Authors:** Mingsong Shi, Zhi Yang, Yuhan Yang, Junxian Chen, Chuandong He, Xiaoan Li

**Affiliations:** 1 National Health Commission Key Laboratory of Nuclear Technology Medical Transformation, Mianyang Central Hospital, School of Medicine, University of Electronic Science and Technology of China, Chengdu, Sichuan, China; 2 Sichuan Provincial Engineering Research Center of Nuclear Medical Equipment Translation and Application, Mianyang Central Hospital, School of Medicine, University of Electronic Science and Technology of China, Chengdu, Sichuan, China; 3 Key Laboratory of General Chemistry of the National Ethnic Affairs Commission, School of Chemistry and Environment, Southwest Minzu University, Chengdu, Sichuan, China; 4 Nuclear Medicine Department, Mianyang Central Hospital, School of Medicine, University of Electronic Science and Technology of China, Chengdu, Sichuan, China; 5 Department of Gastroenterology, Mianyang Central Hospital, School of Medicine, University of Electronic Science and Technology of China, Chengdu, Sichuan, China

**Keywords:** artificial intelligence, fibroblast activation protein, linagliptin, molecular docking, molecular dynamics simulation, tumor microenvironment

## Abstract

Fibroblast activation protein (FAP) is a highly specific biomarker overexpressed in cancer-associated fibroblasts, making it a promising diagnostic target. Developing novel high-affinity molecular probes for FAP-targeted diagnostics remains an active area of research. In this study, an integrated computational pipeline combining generative artificial intelligence, molecular docking, molecular dynamics (MD) simulation, binding energy estimation, and pharmacokinetic predictions was used to design and evaluate novel linagliptin-based compounds as potential FAP-binding analogs. From a preliminary library of 1,016 analogs generated using DeepLigBuilder, eight unique candidates with nine binding models were obtained based on binding affinity predicted using DeepLigBuilder and binding energy based on molecular docking, and four candidates were identified based on dynamic stability assessments and binding energy estimation. Two novel linagliptin-based candidates, Ligand90 and Ligand150, were identified as putative FAP-binding analogs based on ADMET profiling and Lipinski’s Rule of Five compliance. This integrated computational strategy provides a valuable framework to rationally design computationally prioritized candidates for targeting FAP.

## 1 Introduction

Cancer is a major public health challenge worldwide. According to the International Agency for Research on Cancer of the World Health Organization, 19.97 million new cases of cancer and 9.74 million related deaths were registered worldwide in 2022 (Filho et al., 2025). By 2050, the number of new cases may reach 35.281 million and number of deaths may exceed 18.6 million. Despite advances in diagnosis and treatment, challenges such as tumor heterogeneity, drug resistance, and high costs of therapy highlight the need for novel diagnostic and therapeutic strategies (Bai et al., 2023; Ballal et al., 2025; Hua et al., 2025; Siegel et al., 2026; Zheng et al., 2025). Targeted radiopharmaceuticals have emerged as promising tools for the detection of cancer and individualized treatment by specifically binding to key molecular markers in the tumor microenvironment (Zhang S. Q. et al., 2025).

Fibroblast activation protein (FAP) is a type II transmembrane serine protease, which is highly expressed in more than 90% cancer-associated fibroblasts within solid tumors; however, it is minimally expressed in normal tissues, making it an ideal target for tumor diagnosis and therapy (Wang et al., 2025; Wu et al., 2025; Zhao et al., 2024). Radiopharmaceuticals targeting FAP can achieve more sensitive tumor imaging and superior uptake than traditional probes such as ^18^F-FDG. However, most FAP inhibitors (FAPIs), such as FAPI-02, FAPI-04, FAPI-46, OncoFAP glidotin, and KetoFAPI, are derived from UAMC1110 (Supplementary Figure S1; Brzeminski et al., 2025; Galbiati et al., 2026; Köchel et al., 2025; Lindner et al., 2018; Loktev et al., 2019).

**FIGURE 1:**
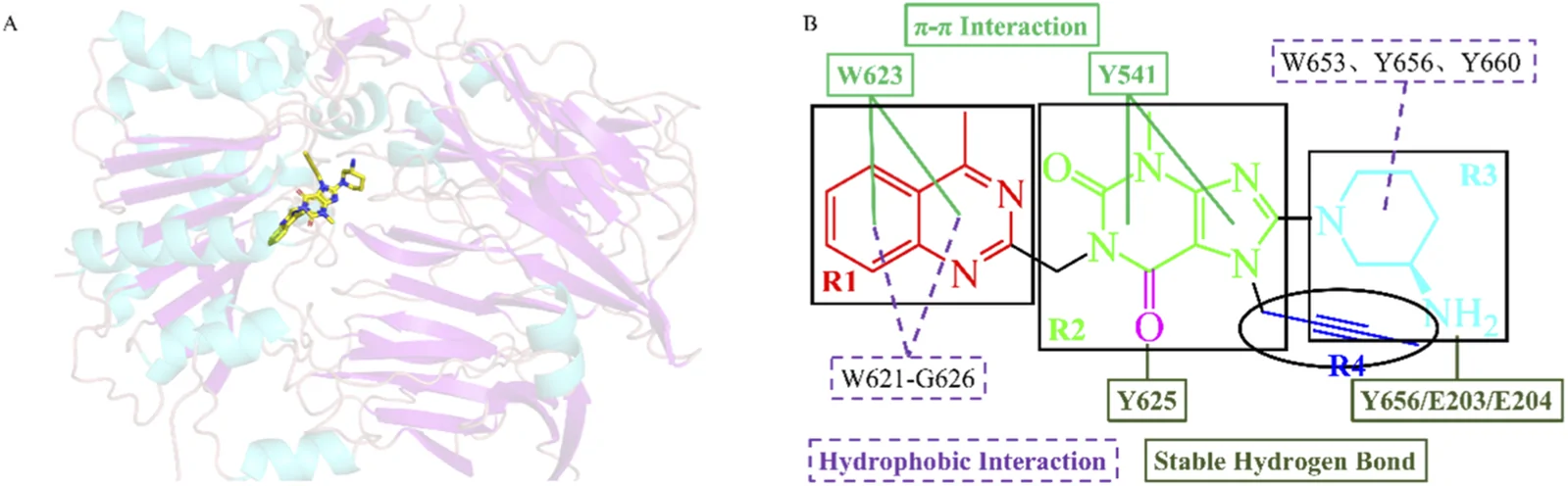
Binding model for linagliptin with FAP. **(A)** Binding model from the crystal structure (PDB ID: 6Y0F) **(B)** Key interaction between linagliptin and FAP (Shi et al., 2024).

Linagliptin, a dipeptidyl peptidase-4 inhibitor, has been approved by the U.S. Food and Drug Administration for treating type-II diabetes (Figure 1; Himmelsbach et al., 2007; Jansen et al., 2014). Linagliptin binds to both dipeptidyl peptidase-4 (half-maximal inhibitory concentration [IC_50_] = 1.4 ± 1.1 nM) and FAP (IC_50_ = 89.9 ± 15.4 nM) (Schnapp et al., 2021; Thomas et al., 2008), and inhibits FAP activity by destroying its dimerization interface (e.g., binding to E203, E204, and Y656). Therefore, rational structural optimization of linagliptin represents a promising strategy to develop high-affinity FAP ligands.

Computational approaches, such as molecular docking, virtual screening, and molecular dynamics (MD) simulation, offer efficient strategies for designing and optimizing novel FAP inhibitors (Meng et al., 2022; Plescia et al., 2022; Zhang Q. Y. et al., 2025). Generative artificial intelligence (AI) has emerged as a transformative modality in medicinal chemistry, enabling *de novo* navigation of expansive chemical spaces and identification of structurally innovative candidates that transcend conventional design paradigms (Kadan et al., 2025; Özçelik et al., 2025; van den Broek et al., 2025; van den Maagdenberg et al., 2025; Abbasi et al., 2026). These methods can accelerate the identification of promising ligands while reducing the cost and time of preclinical studies.

In the present study, a computational design strategy was used to develop novel FAP-targeting linagliptin analogs, which retained key structural features (R2 group, xanthine moiety) of linagliptin (Figure 1), to enable rational ligand optimization.

## 2 Materials and methods

### 2.1 Generative AI

Based on the structure of linagliptin–FAP complex (Shi et al., 2024), the three-dimensional structure of linagliptin was selected as the initial conformation to construct linagliptin analogs using DeepLigBuilder in PharmaMind v.3.12 (Inflygent Pharmaceutical Al Technology Co., Ltd., China) (Li et al., 2021). Novel linagliptin analogs were generated by designing molecules within the FAP activity pocket using the AI model, and then three-dimensional conformations were generated. Binding affinities of the generated analogs were estimated using the AI model in PharmaMind.

### 2.2 Molecular docking

Binding affinities between the linagliptin analogs and human FAP were evaluated using PharmaMind. Classical molecular docking was also used to assess ligand binding affinity. Two box centers were used for docking: one based on the geometric center of the linagliptin analog in DeepLigBuilder and the other based on the geometric center of the initial linagliptin structure. Molecular docking was performed with 100 runs per molecule, and all other parameters were set to default values. Molecular docking files were prepared using AutoDockTools v.1.5.6, and AutoDock v.4.2 was used to generate the grid boxes and perform molecular docking (Morris et al., 2009; Sanner, 1999).

### 2.3 MD simulation

Force fields for linagliptin analogs were constructed using General AMBER Force Field (GAFF) v.2 (He et al., 2020). Ligand structures were optimized and partial atomic charges were calculated at the HF/6-31G* level using Gaussian v.16 (Frisch et al., 2016). The standard AMBER ff19SB force field was applied to human FAP (Tian et al., 2020). Ligand/FAP systems were solvated in a cuboid box of OPC water molecules (approximately 42,000) with a 15 Å buffer, and approximately 102 Na^+^ and 95 Cl^-^ ions were added to control ion concentrations (Izadi et al., 2014). Initial minimization was performed using the steepest descent and conjugate gradient methods while fixing the ligands and FAP. A second minimization step was performed without restrictions using conjugate gradients. Langevin dynamics were used to heat the system from 0 to 300 K over 200 ps. Isotropic position scaling was applied at 300 K and 1 bar for 200 ps, followed by pre-equilibration under the NPT ensemble for 200 ps. Production MD simulations were run for 500 ns using AMBER24 and AMBERTools24 (Case et al., 2023; Case et al., 2024). All trajectories were analyzed using the CPPTRAJ module in AMBERTools24 (Miller et al., 2012; Roe and Cheatham, 2013; Roe and Cheatham, 2018).

### 2.4 Estimation of MM/GBSA-based binding energy

A detailed description of the molecular mechanics generalized born surface area (MM/GBSA) framework is provided elsewhere (Genheden and Ryde, 2015; Honig and Nicholls, 1995; Onufriev and Case, 2019). Relative binding energies between ligands and FAP were estimated using the MM/GBSA framework from the last 200 ns of MD trajectories. The energy contributions of ligand binding were analyzed using the MMPBSA.py tool. Notably, MM/GBSA relies on implicit solvation models and neglects conformational entropy contributions. Therefore, the resulting values are best interpreted as relative metrics for prioritizing candidates rather than absolute thermodynamic measurements. Additional calculation details are provided in the Supplementary Material.

## 3 Results

### 3.1 Generative AI for designing linagliptin analogs

A total of 1,016 linagliptin analogs (named Ligands 1–1016) were generated using generative AI, in which the R2 group (xanthine moiety) of linagliptin was fixed, while the other groups (R1, R3, and R4) were modified. The structure of only one molecule (Ligand878) could not be converted into a canonical SMILES string, precluding automated ChemAudit assessment of its parsability, sanitization, valence, aromaticity, stereochemistry, and connectivity. Manual inspection of its SDF file revealed no structural defects. As Ligand878 was not among the final prioritized candidates, this parsing issue did not affect conclusions. Meanwhile, all linagliptin analogs were predicted to bind the FAP active site, with the xanthine scaffold locked in a conserved native conformation. The minimized affinity values ranged between −7.01 and −3.00; therefore, moderate FAP-targeting potential was predicted. However, this assumption warrants further investigation. In addition, the root mean square deviation (RMSD) between the linagliptin analogs and original linagliptin ranged between 0.01 and 5.44 Å, reflecting similar conformation of ligands in the active site of FAP. The molecular properties of linagliptin analogs are listed in Supplementary Table S1.

### 3.2 Screening for potential candidates targeting FAP through molecular docking

**FIGURE 2:**
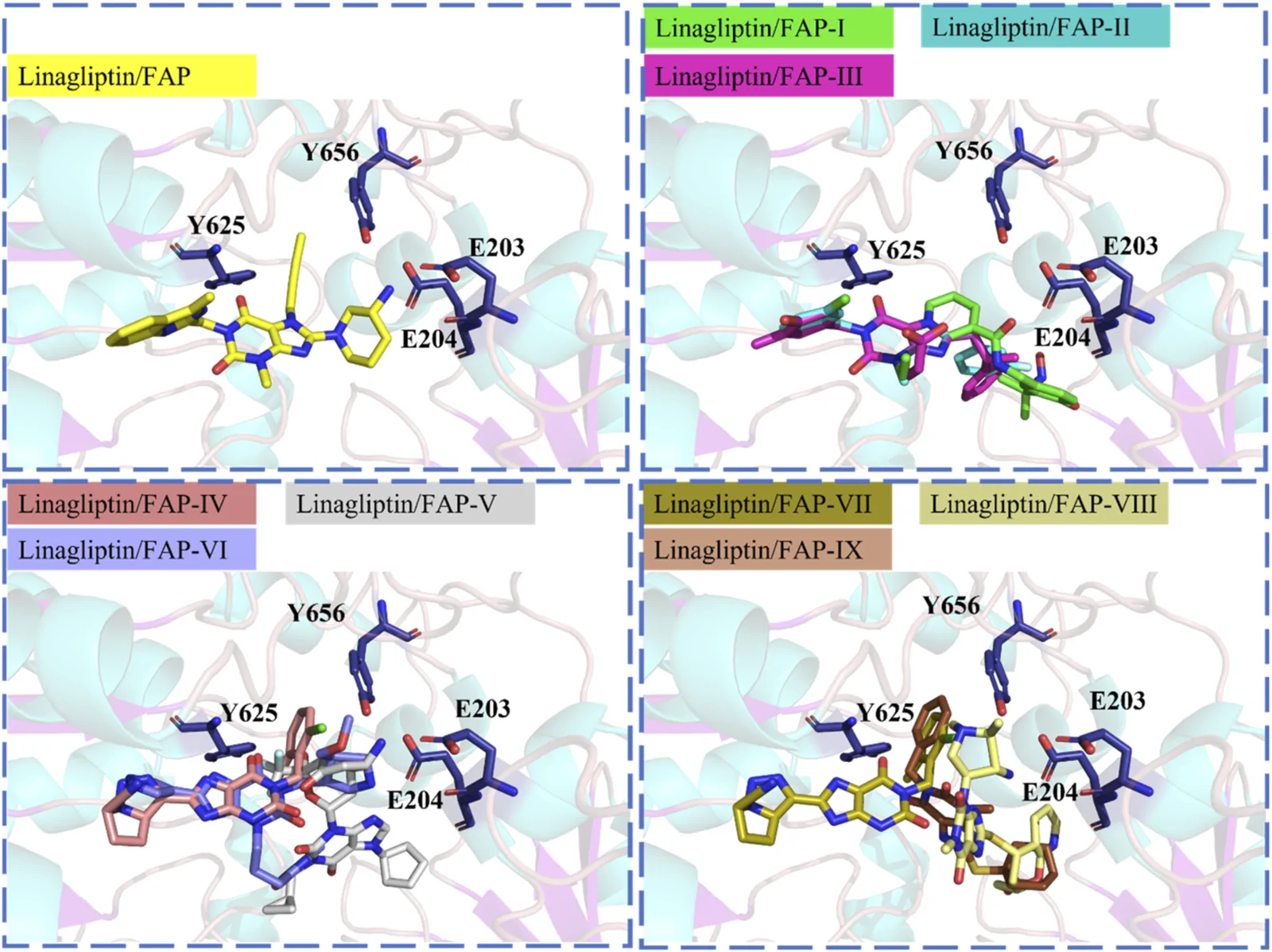
Ligand/FAP complex systems obtained by screening.

To properly evaluate the binding affinity of linagliptin analogs for FAP, redocking was performed for native linagliptin, and docking was performed for the linagliptin analogs. Native linagliptin was re-docked into the FAP-binding pocket following standard procedure to validate the reliability of the docking protocol. The lowest binding-energy pose (−11.21 kcal/mol) showed an RMSD of 0.76 Å with the original pose of the initial linagliptin/FAP complex (Supplementary Figure S3), indicating that the docking protocol reliably reproduced the original binding mode. To comprehensively evaluate the binding potential, two distinct AutoDock strategies were used based on different definitions of the search space. In AutoDock-I, the docking box was placed on the geometric center of the original linagliptin structure, focusing on the conserved binding pocket. Conversely, AutoDock-II placed the docking box on the geometric center of each individual ligand, allowing for the exploration of ligand-specific binding modes. This dual approach ensured balanced assessments of both canonical and novel binding conformations. The binding energies from AutoDock-I ranged between −9.87 and −4.32 kcal/mol (Figure 2), whereas those from AutoDock-II ranged between −10.31 and −4.34 kcal/mol. Notably, a significant difference was observed in the binding-affinity score between the AI model (DeepLigBuilder) and that obtained through classical molecular docking (AutoDock), which resulted from differences in scoring functions and sampling methods. This difference primarily resulted from differences in conformational sampling constraints. DeepLigBuilder enforced strict topological rigidity, preserving the R2 (xanthine) moiety of linagliptin in its native pose, which limited conformational exploration (Supplementary Figures S4–S13). Conversely, AutoDock explored a relatively broad conformational space, allowing the ligands to sample alternative binding modes within the expansive active site of FAP. Furthermore, different definitions of the docking-box centers also led to distinct energy profiles, probably owing to the large active site of FAP with multiple binding conformations.

Based on the AI-based binding scores, the top three ligands (Ligand1, Ligand2, and Ligand3) exhibited the highest affinities, with scores of −7.01, −6.96, and −6.51, respectively. In comparison, AutoDock-I identified Ligand150 (−9.87 kcal/mol), Ligand556 (−9.81 kcal/mol), and Ligand427 (−9.75 kcal/mol) as having the strongest binding energies. When the docking box center was shifted to the geometric center of the ligand (AutoDock-II), Ligand150 (−10.31 kcal/mol), Ligand141 (−10.09 kcal/mol), and Ligand90 (−10.06 kcal/mol) emerged as the top candidates. These eight compounds with nine binding models (the top three compounds from AI-based binding scores, AutoDock-I, and AutoDock-II, respectively) were selected as potential FAP-binding candidates for further investigation because of their favorable binding energies.

In the binding mode illustrated in Figure 2, these analogs were situated on one side of the FAP pocket, leaving a substantial solvent-accessible region. This observation suggests that linagliptin analogs may occupy alternative binding sites within the active pocket.

### 3.3 Stability assessment of the candidates bound to FAP using MD simulation

The systems investigated in the preceding studies were designated as follows: Ligand/FAP-I (MolBuilder, Top1, and Ligand1), Ligand/FAP-II (MolBuilder, Top2, and Ligand2), Ligand/FAP-III (MolBuilder, Top3, and Ligand3), Ligand/FAP-IV (AutoDock-I, Top1, and Ligand150), Ligand/FAP-V (AutoDock-I, Top2, and Ligand556), Ligand/FAP-VI (AutoDock-I, Top3, and Ligand427), Ligand/FAP-VII (AutoDock-II, Top1, and Ligand150), Ligand/FAP-VIII (AutoDock-II, Top2, and Ligand141), and Ligand/FAP-IX (AutoDock-II, Top3, and Ligand90).

**FIGURE 3:**
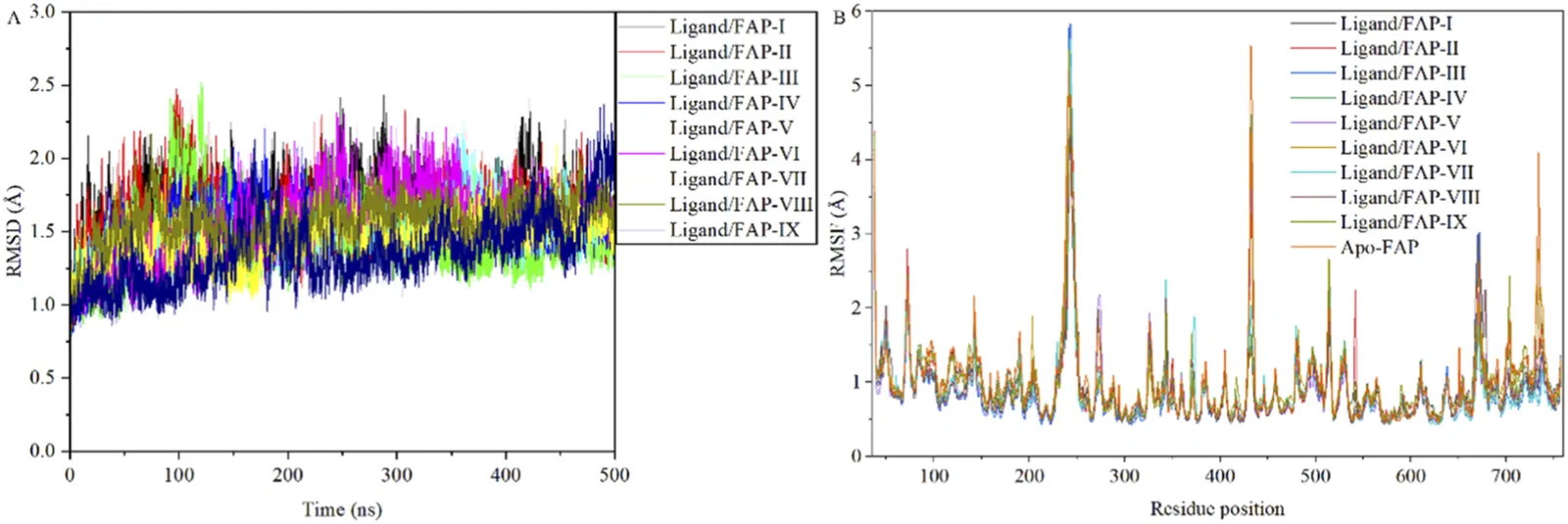
System stability of ligands binding to FAP. **(A)** Root mean square deviation (RMSD) of backbone heavy atoms of human FAP during the 500-ns MD simulation. **(B)** Variation in root mean square fluctuation (RMSF) for Cα atoms of human FAP for Ligand/FAP and apo-FAP systems throughout the 500-ns MD simulations.

MD simulations were performed for 500 ns for each complex system. The RMSD values of FAP indicated that all systems were stable after 100 ns, except for the Ligand/FAP-III system (Figure 3; Supplementary Figure S14; Supplementary Table S2). A large fluctuation at approximately 240 ns was observed for Ligand/FAP-III, which may be due to a conformational change of the solvation exposure domain (Supplementary Figure S15). However, the RMSD values of the ligands indicated that some of them failed to retain the initial binding pose. For example, the ligands generated by DeepLigBuilder failed to retain their initial scaffold conformation (constrained by the generative model) during MD simulations, probably owing to the fact that the R2 group of linagliptin was fixed for generating novel ligands based on AI using MolBuilder. Meanwhile, modification of the other groups (such as R1, R3, and R4) may change the binding mode between ligands and FAP. Preserving the binding mode of the parental linagliptin scaffold may necessitate retaining additional key moieties, such as the R2 and R3 groups, during AI-driven optimization. This observation indicates that the original ligand can be partially modified, and a single region (such as one of R1, R3, and R4 groups) may be optimized using MolBuilder to retain the same binding mode. In addition, the conformations from AutoDock were stable, except for Ligand/FAP-VI. The binding site of Ligand427 changed from the initial conformation to a nearby site after 250 ns for the Ligand/FAP-VI system (Supplementary Figure S16). In general, stabilizing the binding mode of the complex system constructed by MolBuilder was difficult because of large numbers of modified groups, whereas the complex system reconstructed by AutoDock was stable in MD simulations. This phenomenon was supported by analyses of surface area and gyration radius (Supplementary Figures S17, S18). Therefore, the binding mode must be reconsidered if the ligand is modified with a relatively large structure.

Root mean square fluctuation (RMSF) was calculated to evaluate the residue-level flexibility of the ligand-bound FAP and apo-FAP systems (Figure 3). Ligand binding resulted in reduced RMSF values in specific loop regions compared to the apo state, suggesting restricted local mobility upon complex formation. In addition, the β-propeller domain 4, located at the FAP dimer interface, exhibited substantial fluctuations during simulations. Frames at 100, 200, 300, 400, and 500 ns were extracted to analyze fluctuations in the β-propeller domain four region (Supplementary Figures S19–S27). Meanwhile, ligand binding had minimal impact on the overall FAP structure, as revealed by comparison with the apo-FAP system without any ligand (Supplementary Figure S28). However, residues 429–436 of apo-FAP exhibited relatively high fluctuations and were primarily exposed to the solvent, away from the binding pocket. Consequently, the ligand conformation obtained via AutoDock remained stable during MD simulations, whereas that generated using MolBuilder was unstable, undergoing substantial structural modifications.

### 3.4 Affinity of potential FAP-binding candidates based on binding energy estimation

The relative binding energies (estimated via MM/GBSA) of the potential ligands ranged between −31.54 and −17.87 kcal/mol (Tables 1; Supplementary Tables S3–S11), indicating thermodynamically favorable binding affinity between FAP and the ligands. However, their binding ability was less than that of linagliptin, probably owing to a strong interaction between the R3 group (not present in the ligands) of linagliptin and three residues (Y656, E203, and E204) of FAP through hydrogen bonding. In addition, electrostatic (−44.40 to −5.96 kcal/mol) and Van der Waals (−49.73 to −32.62 kcal/mol) interaction energies indicated that the latter primarily contributed to ligand binding with FAP. Meanwhile, $E_{\text{polar}}$ was consistently positive (20.26–69.28 kcal/mol), representing the unfavorable desolvation penalty required to displace water molecules from the binding interface. While the solvent effect opposes binding, strong favorable contributions from Van der Waals and electrostatic interactions outweigh this cost, resulting in a net negative $\Delta E_{\text{bind}}^{\text{cal}}$ . The Ligand/FAP-IV, Ligand/FAP-V, Ligand/FAP-VII, Ligand/FAP-VIII, and Ligand/FAP-IX systems exhibited more favorable (more negative) binding energies than −25.00 kcal/mol. Meanwhile, a large difference in binding energies was observed between MD simulation and MolBuilder. Overall, Ligand90, Ligand141, Ligand150, and Ligand556 (with binding energies < −25.00 kcal/mol) were identified as putative compounds binding with FAP.

**TABLE 1:** Binding energies ( $\Delta E_{\text{bind}}^{\text{cal}}$ ) for ligand/FAP complex systems.

Energy (kcal/mol)	Ligand/FAP-I	Ligand/FAP-II	Ligand/FAP-III	Ligand/FAP-IV	Ligand/FAP-V
$E_{\text{vdW}}$	−32.62	−36.78	−35.82	−47.16	−49.73
$E_{\text{ele}}$	−14.89	−10.19	−18.23	−7.45	−21.41
$E_{\text{polar}}$	32.26	28.08	38.89	28.04	50.41
$E_{\text{nonpolar}}$	−4.41	−4.70	−3.57	−4.96	−6.32
$E_{\text{gas}}$	−47.50	−46.96	−54.06	−54.61	−71.13
$E_{\text{solv}}$	27.86	23.37	35.31	23.08	44.08
$\Delta E_{\text{bind}}^{\text{cal}}$	−19.64	−23.59	−18.74	−31.53	−27.04

Energy (kcal/mol)	Ligand/FAP-VI	Ligand/FAP-VII	Ligand/FAP-VIII	Ligand/FAP-IX
$E_{\text{vdW}}$	−42.03	−41.42	−47.13	−41.42
$E_{\text{ele}}$	−11.93	−5.96	−44.40	−18.00
$E_{\text{polar}}$	41.40	20.26	69.28	37.98
$E_{\text{nonpolar}}$	−5.32	−4.42	−6.25	−4.92
$E_{\text{gas}}$	−53.96	−47.37	−91.53	−59.41
$E_{\text{solv}}$	36.09	15.84	63.03	33.06
$\Delta E_{\text{bind}}^{\text{cal}}$	−17.87	−31.54	−28.50	−26.35

Binding energies ( $\Delta E_{\text{bind}}^{\text{cal}}$ ) of ligand/FAP systems and decomposition to electrostatic interaction ( $E_{\text{ele}}$ ), Van der Waals interaction ( $E_{\text{vdW}}$ ), polar solvation free energies ( $E_{\text{polar}}$ ), and nonpolar solvation free energies ( $E_{\text{nonpolar}}$ ). E_gas_, molecular mechanical energies summarized from $E_{\text{vdW}}$ and $E_{\text{ele}}$ ; E_solv_, solvation free energy divided into $E_{\text{polar}}$ and $E_{\text{nonpolar}}$ . Uncertainties were calculated as the root mean square error for all frames extracted from the trajectories and are presented in Supplementary Tables S23–S11.

### 3.5 Pharmacokinetic and toxicological profiles of putative FAP-binding analogs

Drug-like properties of Ligand90, Ligand141, Ligand150, and Ligand556 were predicted using SwissADME (Supplementary Figure S29; Daina et al., 2017). Ligand90 and Ligand150 followed the Lipinski’s rule of Five (Table 2). Ligand141 and Ligand556 exhibited a single violation of Lipinski’s Rule of Five. Water solubility of the four compounds was directly estimated from the molecular structure, and all compounds excluding Ligand90 were water-soluble. Compounds exhibiting a bioavailability score of 0.55 were prioritized to ensure favorable physicochemical properties (e.g., molecular weight and lipophilicity) conducive to efficient distribution and tissue penetration that are the key criteria for effective probe development. Synthetic accessibility analysis indicated that these compounds could be readily synthesized. Finally, Ligand90 and Ligand150 (followed Lipinski’s Rule of Five) were identified as computationally prioritized candidates targeting FAP.

**TABLE 2:** Predicted ADME and drug-like parameters of potential ligands.

Ligand	Ligand90	Ligand141	Ligand150	Ligand556	Linagliptin
MW (g/mol)	410.49	473.55	422.83	511.49	473.55
nRB	4	4	3	5	4
nHBA	4	8	6	10	5
nHBD	1	3	2	2	1
iLOGP	2.83	2.48	1.84	2.39	3.73
TPSA	115.05	169.59	138.04	134.37	118.48
Lipinski	0	1	0	1	0
ESOL class	Moderately soluble	Soluble	Soluble	Soluble	Moderately soluble
BS	0.55	0.55	0.55	0.55	0.55
SA	4.61	5.18	3.7	5.31	4.44

MW, molecular weight (g/mol); nRB, number of rotatable bonds; nHBA, number of hydrogen bond acceptors; nHBD, number of hydrogen bond donors; iLOGP, octanol/water partition coefficient; TPSA, topological polar surface area; ESOL, estimated aqueous solubility; BS, bioavailability score; SA, synthetic accessibility; ADME, absorption, distribution, metabolism, and excretion.

## 4 Discussion

FAP has emerged as a pivotal diagnostic target in oncology because of its selective and high expression in cancer-associated fibroblasts of solid tumors. Clinically, ^68^Ga-labeled FAP-targeted radiopharmaceuticals, such as ^68^Ga-FAPI-04 and ^68^Ga-FAPI-46, have shown excellent imaging performance (Alcin et al., 2023; Hess et al., 2025; Meng et al., 2025; Watanabe et al., 2024; Yu et al., 2026). However, several first-generation FAP-targeted probes exhibit suboptimal pharmacokinetics, including rapid clearance and non-target uptake, which limit the imaging windows and tumor-to-background ratios (Altmann et al., 2021; Jiang et al., 2021). Improving probe affinity and prolonging tumor retention are essential for detecting lesions with low or heterogeneous FAP expression, and novel scaffolds beyond quinoline-based inhibitors are needed to achieve these goals.

Linagliptin, a clinically approved dipeptidyl peptidase-4 inhibitor for type 2 diabetes, has recently garnered attention owing to its interaction with FAP (Jansen et al., 2014; Schnapp et al., 2021; Thomas et al., 2008). Its established safety profile and favorable drug-like properties make linagliptin a valuable pharmacological starting point for designing FAP-targeted probes. Rationally designed linagliptin analogs may improve binding affinity with FAP and optimize pharmacokinetic properties including lipophilicity and tumor retention.

**FIGURE 4:**
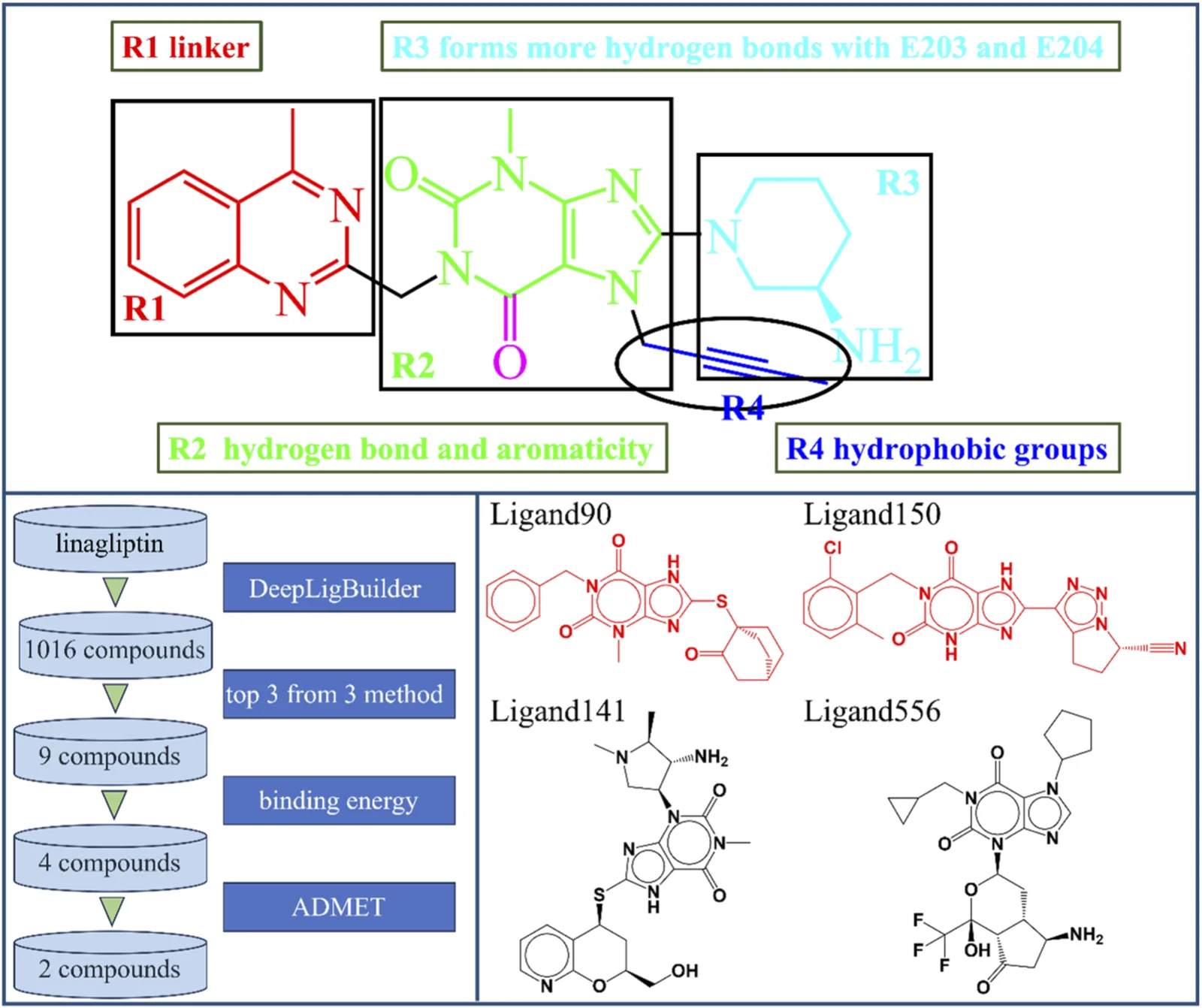
Virtual screening pipeline from linagliptin analogs targeting FAP. The 1,016 compounds were generated from the fixed R2 group (xanthine moiety) of linagliptin, using DeepLigBuilder. The top three compounds based on binding affinity were selected using DeepLigBuilder, AutoDock-I, and AutoDock-II. The binding energies calculated by MM/GBSA were < −25 kcal/mol, revealing potential FAP-targeting candidates. Two compounds (Ligand90 and Ligand150) followed the Lipinski’s Rule of Five.

In the present study, 1,016 linagliptin analogs were designed based on generative AI using DeepLigBuilder in MolBuilder. Eight candidate ligands (Ligand1, Ligand2, Ligand3, Ligand90, Ligand141, Ligand150, Ligand427, and Ligand556) were predicted to have high binding affinities using generative AI and molecular docking (Figure 4). Ligand90, Ligand141, Ligand150, and Ligand556 were identified as putative compounds binding with FAP, based on dynamic stability assessments and binding energy estimation (with binding energy < −25.00 kcal/mol). Ligand90 and Ligand150 showed favorable ADMET profiles and complied with Lipinski’s Rule of Five, highlighting their potential as the putative FAP-binding analogs.

MD simulations revealed that some AI-generated complexes were unstable, whereas the systems selected via docking exhibited stable binding. This highlights the importance of dynamic validation for confirming plausible binding modes and eliminating false positives, thereby emphasizing that routine evaluation of conformational dynamics is essential for developing small FAP-targeted probes.

Notably, the conformational rigidity imposed by generative DeepLigBuilder, specifically the constraint of preserving the R2 (xanthine) moiety of linagliptin in its native pose (Supplementary Figure S30), probably contributed to the binding instability observed in MD simulations. While DeepLigBuilder maintained scaffold similarity to linagliptin, extensive modifications to peripheral groups failed to fully account for protein-induced conformational adaptation. By contrast, AutoDock performed conformational searches within the FAP-binding pocket and identified conformations that were significantly different from the original linagliptin conformation; however, the conformation of ligands such as Ligand90 and Ligand150 was dynamically stable (Supplementary Figure S31). This observation reveals a critical methodological gap, indicating that DeepLigBuilder-generated molecules often lack the conformational plasticity required for optimal target engagement. These findings underscore that DeepLigBuilder-derived candidates, particularly those with extensive group modifications, necessitate rigorous multi-level validation. Dynamic simulations and experimental assays are essential to filter out false positives arising from conformational artifacts. However, comprehensive validation regarding the source of these conformational discrepancies is currently lacking. Additionally, whether this observed instability is unique to the FAP binding site or generalizable to other protein targets and diverse scaffold modifications is unclear. Therefore, rigorous benchmarking across multiple biological systems is required to fully validate the physical plausibility of AI-generated ligand conformations.

Despite these promising *in silico* findings, this study has several limitations. Experimental validation of the predicted ligands is lacking, and developing ^68^Ga-labeled FAP-targeted radiopharmaceuticals based on linagliptin analogs has not been addressed. Future studies should focus on synthesizing promising analogs, evaluating their activity and selectivity *in vitro* and *in vivo*, and assessing their potential as diagnostic radiopharmaceuticals. Meanwhile, one group of linagliptin (R1, R3, or R4) may be replaced using the AI model and compared with molecular docking results to evaluate the plausible binding poses.

In conclusion, this study establishes a computational pipeline for designing linagliptin analogs as putative FAP-binding candidates. For example, Ligand90 and Ligand150 were identified as computationally prioritized candidates targeting FAP. By integrating AI-driven ligand design, molecular docking, and MD simulations, this study provides a framework for accelerating the discovery of high-affinity FAP-binding candidates for further synthesis and validation.

## Data Availability

The original contributions presented in the study are included in the article/Supplementary Material, further inquiries can be directed to the corresponding authors.
