# Supplementary material for: Designing novel linagliptin analogs through combined generative artificial intelligence, molecular docking, molecular dynamics simulation, and binding energy estimation for targeting fibroblast activation protein
Source: Front Chem. 2026 Sep 9;14:1848970. doi: 10.3389/fchem.2026.1848970 (PMC13597733; doi:10.3389/fchem.2026.1848970)
Supplement: Supplementary file 1 [file DataSheet2.pdf]

Designing Novel Linagliptin Analogs Through Combined Generative Artificial Intelligence, Molecular Docking, Molecular Dynamics Simulation, and Binding Energy Estimation for Targeting Fibroblast Activation Protein

Mingsong Shi[1,2], Zhi Yang[1], Yuhan Yang[1], Junxian Chen[3], Chuandong He[4*], Xiaoan Li[1,5*]

[1]National Health Commission Key Laboratory of Nuclear Technology Medical Transformation, Mianyang Central Hospital, School of Medicine, University of Electronic Science and Technology of China, Chengdu, Sichuan, 610054, China

[2]Sichuan Provincial Engineering Research Center of Nuclear Medical Equipment Translation and Application, Mianyang Central Hospital, School of Medicine, University of Electronic Science and Technology of China, Chengdu, Sichuan, 610054, China

[3]Key Laboratory of General Chemistry of the National Ethnic Affairs Commission, School of Chemistry and Environment, Southwest Minzu University, Chengdu, Sichuan, 610041, China

[4]Nuclear Medicine Department, Mianyang Central Hospital, School of Medicine, University of Electronic Science and Technology of China, Chengdu, Sichuan, 610054, China

[5]Department of Gastroenterology, Mianyang Central Hospital, School of Medicine, University of Electronic Science and Technology of China, Chengdu, Sichuan, 610054, China

*** Correspondence:**
Chuandong He
hecd735@163.com

Xiaoan Li
lixiaoan@sc-mch.cn

Contents

Molecular docking S1

MD simulations..S1

MM/GBSA-based binding energy S2

Figure S1 Structures for main FAP inhibitors...S8

Figure S2 Structure of Ligand878...S9

Figure S3 Docking results for original linagliptin structure with FAP S10

Figure S4 Initial linagliptin/FAP complex system S11

Figure S5 Initial ligand/FAP-I complex system...S12

Figure S6 Initial ligand/FAP-II complex system S13

Figure S7 Initial ligand/FAP-III complex system..S14

Figure S8 Initial ligand/FAP-IV complex system..S15

Figure S9 Initial ligand/FAP-V complex system S16

Figure S10 Initial ligand/FAP-VI complex system..S17

Figure S11 Initial ligand/FAP-VII complex system...S18

Figure S12 Initial ligand/FAP-VIII complex system S19

Figure S13 Initial ligand/FAP-IX complex system..S20

Figure S14 Root mean square deviation (RMSD) of the backbone heavy atoms in human FAP and non-hydrogen atoms of the ligands during the 500-ns MD simulations of the Ligand/FAP complex systems..S21

Figure S15 Snapshots of the Ligand/FAP-III complex during the MD simulation at 230-260 ns S22

Figure S16 Snapshots of the Ligand/FAP-VI complex during the MD simulation at 240-360 ns S23

Figure S17 Surface area for ligand binding with FAP S24

Figure S18 Gyration radius for ligand binding with FAP..S25

Figure S19 Snapshots of the Ligand/FAP-I complex during the MD simulation at 100, 200, 300, 400, and 500 ns S26

Figure S20 Snapshots of the Ligand/FAP-II complex during the MD simulation at 100, 200, 300, 400, and 500 ns S27

Figure S21 Snapshots of the Ligand/FAP-III complex during the MD simulation at 100, 200, 300, 400, and 500 ns S28

Figure S22 Snapshots of the Ligand/FAP-IV complex during the MD simulation at 100, 200, 300, 400, and 500 ns S29

Figure S23 Snapshots of the Ligand/FAP-V complex during the MD simulation at 100, 200, 300, 400, and 500 ns S30

Figure S24 Snapshots of the Ligand/FAP-VI complex during the MD simulation at 100, 200, 300, 400, and 500 ns S31

Figure S25 Snapshots of the Ligand/FAP-VII complex during the MD simulation at 100, 200, 300, 400, and 500 ns S32

Figure S26 Snapshots of the Ligand/FAP-VIII complex during the MD simulation at 100, 200, 300, 400, and 500 ns S33

Figure S27 Snapshots of the Ligand/FAP-IX complex during the MD simulation at 100, 200, 300, 400, and 500 ns S34

Figure S28 RMSF variations for Cα atom of human FAP for ligand/FAP and Apo-FAP systems from the 500 ns MD simulation S35

Figure S29 Predicted ADME properties and drug-likeness profiles for the top-ranked linagliptin analogs S36

Figure S30 Preservation of the R2 (xanthine) scaffold in AI-generated ligands S37

Figure S31 Aligned conformations of Ligand90 and Ligand150 using AI and molecular docking...S38

Table S1 Score value for linagliptin analogues...S39

Table S2 RMSD value for the ligand/FAP complex systems during 500-ns MD simulations S40

Table S3 Binding energies ($\Delta\mathbf{E}_{\mathbf{bind}}^{\mathbf{cal}}$) for Ligand/FAP-I complex system S41

Table S4 Binding energies ($\Delta\mathbf{E}_{\mathbf{bind}}^{\mathbf{cal}}$) for Ligand/FAP-II complex system..................S42

Table S5 Binding energies ($\Delta\mathbf{E}_{\mathbf{bind}}^{\mathbf{cal}}$) for Ligand/FAP-III complex system S43

Table S6 Binding energies ($\Delta\mathbf{E}_{\mathbf{bind}}^{\mathbf{cal}}$) for Ligand/FAP-IV complex system S44

Table S7 Binding energies ($\Delta\mathbf{E}_{\mathbf{bind}}^{\mathbf{cal}}$) for Ligand/FAP-V complex system S45

Table S8 Binding energies ($\Delta\mathbf{E}_{\mathbf{bind}}^{\mathbf{cal}}$) for Ligand/FAP-VI complex system S46

Table S9 Binding energies ($\Delta\mathbf{E}_{\mathbf{bind}}^{\mathbf{cal}}$) for Ligand/FAP-VII complex system...............S47

Table S10 Binding energies ($\Delta\mathbf{E}_{\mathbf{bind}}^{\mathbf{cal}}$) for Ligand/FAP-VIII complex system S48

Table S11 Binding energies ($\Delta\mathbf{E}_{\mathbf{bind}}^{\mathbf{cal}}$) for Ligand/FAP-IX complex system S49

Molecular docking

Based on the linagliptin/FAP complex obtained in a previous report (Shi et al., 2024), the receptor–ligand complex structure was utilized for molecular docking simulations. FAP structure was pretreated through the AutoDockTools v.1.5.6 (Sanner, 1999), including Gasteiger charge, adjustment of unreasonable atomic overlap, and merged non-polar hydrogen atoms. The structures of linagliptin and other ligands were obtained without hydrogen atoms using DeepLigBuilder in PharmaMind v.3.12 (Inflygent Pharmaceutical Al Technology Co., Ltd. China) (Li et al., 2021). Polar hydrogen atoms were added to the linagliptin analogues using AutoDockTools v.1.5.6. A grid map with 0.375-Å grid spacing was generated using AutoGrid based on the geometric center of the linagliptin analog in DeepLigBuilder or the initial linagliptin structure from original linagliptin/FAP complex. One hundred docking conformations were generated for each system using the Lamarckian genetic algorithm implemented in AutoDock v4.2 (Morris et al., 2009). Finally, the conformation exhibiting the lowest binding energy for each docking system was taken into consideration.

MD simulations

The ligand conformation with the lowest predicted binding energy for each of the nine binding systems was selected for MD simulations, which also included the FAP structure from the initial linagliptin/FAP complex. To accurately parameterize linagliptin and its analogs, the General AMBER Force Field (GAFF2) (Wang et al., 2004) was employed. All hydrogen atoms were added to the ligands using the Antechamber software based on the conformation from AutoDock (with polar hydrogen atoms) or PharmaMind (without hydrogen atoms). The geometric structure was optimized at the HF/6-31G* level of theory using Gaussian v.16 (Frisch et al., 2016). To obtain partial atomic charges, the restrained electrostatic potential protocol (Bayly et al., 1993) was used at the HF/6-31G* level of theory. The force field parameters were generated using the Antechamber module. Meanwhile, AMBER ff19SB force field (Tian et al., 2020) was used to create topology parameters of FAP. The protonation state of every residue in FAP was determined at physiological pH (pH = 7.4) using the H++ online service (Bashford and Karplus, 1990; Gordon et al., 2005; Myers et al., 2006; Anandakrishnan et al., 2012). First, complex systems were dissolved in OPC water (Izadi et al., 2014) with cuboid box, and the total box size was approximately 108 Å ×

117 Å × 121 Å. The ff19SB pairs best with the relatively accurate water model OPC (Tian et al., 2020). The systems were neutralized using sodium (Na^+) and chloride (Cl^-) ions. The periodic boundary conditions were used to avoid edge effects, and a cut-off radius of 12 Å was used for van der Waals interactions. The particle mesh Ewald (PME) algorithm (Darden et al., 1993) was used in calculating long-range electrostatic interactions. The SHAKE algorithm (Ryckaert et al., 1977) was used to constrain the covalent bond involved with hydrogen atoms. To reduce the effect of unfavorable interactions produced by solvents and ions, the system was subjected to the 9000 steps of steepest descent method followed by 1000-step conjugate gradient while fixing all solute molecules at the initial position. Then, the 10000-step conjugate gradient method was used to optimize the whole system including solute and solvent molecules. After the first two-step minimization of the system, the overall system temperature was increased from 0 to 300 K in 200 ps using Langevin dynamics (Martyna et al., 1994; Feller et al., 1995) with the collision frequency g = 2.0 ps^{-1}. The pressure was kept at 1 bar for 200 ps with isotropic position scaling (Berendsen et al., 1984), and the temperature was maintained at 300 K. After that, the system was equilibrated at 300 K and 1 bar within the NPT (isothermal–isobaric) ensemble for 200 ps. Subsequently, the entire system underwent a 500-ns MD simulation for final data collection and analyses. The integration-step size was set to 2 fs throughout the simulation. The random seed was based on the current date and time for every ns simulation in 500-ns MD simulations. All dynamics were performed using the CUDA version of PMEMD in AMBER v.24 (Case et al., 2024). The *CPPTRAJ* module (Roe and Cheatham, 2013; 2018) was used to analyze the data of MD trajectories.

MM/GBSA-based binding energy

In addition to qualitative analysis of ligand/protein binding, quantitative analysis is equally important in determining the binding affinity between ligands and proteins. Several methods, such as molecular mechanics/Poisson Boltzmann (or generalized Born) surface area (MM/PBSA or MM/GBSA) (Srinivasan et al., 1998; Lee et al., 2004), solvated interaction energy (Naim et al., 2007), linear interaction energy (Aqvist et al., 1994; Bren et al., 2006; Perdih et al., 2009), free energy pathway method (Gilson and Zhou, 2007), and linear response approximation (Lee et al., 1992), have been developed to estimate the absolute binding energy between inhibitors and their target proteins. The

MM/GBSA approach is a timesaving and efficient method to evaluate the binding energy between inhibitors and proteins (Cheng et al., 2018; King et al., 2021; Shi et al., 2021; Shi et al., 2022; Shirvani and Fassihi, 2022). In this study, only a short description of the calculation of the binding energy (ΔE_{bind}^{cal}) from the MM/GBSA method is provided in the following formulae:

$$\Delta E_{bind}^{cal} = E_{complex} - E_{protein} - E_{ligand} \quad (1)$$

$$E = E_{gas} + E_{sol} \quad (2)$$

$$E_{gas} = E_{int} + E_{vdW} + E_{ele} \quad (3)$$

$$E_{sol} = E_{polar} + E_{nonpolar} \quad (4)$$

$$E_{nonpolar} = \gamma \cdot SA + b \quad (5)$$

$E_{complex}$, $E_{protein}$, and E_{ligand} denote the free energies of ligand/FAP, FAP, and ligand, respectively. E can be decomposed into $E_{gas} + E_{sol}$. The molecular mechanical energies (E_{gas}) can also be summarized from the intramolecular energy (E_{int}), van der Waals forces (E_{vdW}), and electrostatic forces (E_{ele}). Meanwhile, the contributions of E_{int}, E_{vdW}, and E_{ele} can be obtained through the statistical average based on molecular mechanics. In addition, solvation free energy (E_{sol}) can be divided into polar solvation (E_{polar}) and nonpolar solvation energies ($E_{nonpolar}$). $E_{nonpolar}$ is obtained from the favorable van der Waals interactions between the solute and solvent and the unfavorable cost of surface formation. $E_{nonpolar}$ can be calculated using equation (5), where $\gamma = 0.0072$ kcal/Å^2 and $b = 0.0$ kcal/mol. The linear combination of pairwise overlaps (LCPO) method (Weiser et al., 1999) was employed to estimate the solvent-accessible surface area (*SA*). However, the E_{polar} contribution was calculated from the GB equation (Still et al., 1990; Srinivasan et al., 1999). The dielectric constant for the ligand/protein and water were set to 1 and 80, respectively. Thousand snapshots were extracted from the last 200 ns MD trajectory to calculate the statistical average of the MM/GBSA method.

Reference

Anandakrishnan, R., Aguilar, B., and Onufriev, A. V. (2012). H++3.0: automating pK prediction and the preparation of biomolecular structures for atomistic molecular

modeling and simulations. *Nucleic Acids Research* 40, W537-W541.
Aqvist, J., Medina, C., and Samuelsson, J. E. (1994). A new method for predicting binding affinity in computer-aided drug design. *Protein Engineering* 7, 385-391.
Bashford, D., and Karplus, M. (1990). pKa of ionizable groups in proteins: Atomic detail from a continuum electrostatic model. *Biochemistry* 29, 10219-10225.
Bayly, C. I., Cieplak, P., Cornell, W. D., and Kollman, P. A. (1993). A well-behaved electrostatic potential based method using charge restraints for deriving atomic charges: the RESP model. *Journal of Physical Chemistry* 97, 10269-10280.
Berendsen, H. J. C., Postma, J. P. M., Vangunsteren, W. F., Dinola, A., and Haak, J. R. (1984). Molecular dynamics with coupling to an external bath. *Journal of Chemical Physics* 81, 3684-3690.
Bren, U., Martinek, V., and Florian, J. (2006). Free energy simulations of uncatalyzed DNA replication fidelity: Structure and stability of T center dot G and dTTP center dot G terminal DNA mismatches flanked by a single dangling nucleotide. *Journal of Physical Chemistry B* 110, 10557-10566.
Case, D. A., Aktulga, H. M., Belfon, K., Ben-Shalom, I. Y., Berryman, J. T., Brozell, S. R., Cerutti, D. S., Cheatham, T. E., Iii, G. A. C., Cruzeiro, V. W. D., Darden, T. A., Forouzesh, N., Ghazimirsaeed, M., Giambaşu, G., Giese, T., Gilson, M. K., Gohlke, H., Goetz, A. W., Harris, J., Huang, Z., Izadi, S., Izmailov, S. A., Kasavajhala, K., Kaymak, M. C., Kovalenko, A., Kurtzman, T., Lee, T. S., Li, P., Li, Z., Lin, C., Liu, J., Luchko, T., Luo, R., Machado, M., Manathunga, M., Merz, K. M., Miao, Y., Mikhailovskii, O., Monard, G., Nguyen, H., O'hearn, K. A., Onufriev, A., Pan, F., Pantano, S., Rahnamoun, A., Roe, D. R., Roitberg, A., Sagui, C., Schott-Verdugo, S., Shajan, A., Shen, J., Simmerling, C. L., Skrynnikov, N. R., Smith, J., Swails, J., Walker, R. C., Wang, J., Wang, J., Wu, X., Wu, Y., Xiong, Y., Xue, Y., York, D. M., Zhao, C., Zhu, Q., and Kollman, P. A. (2024). Amber 2024, University of California, San Francisco.
Cheng, P., Li, J. J., Wang, J., Zhang, X. Y., and Zhai, H. L. (2018). Investigations of FAK inhibitors: a combination of 3D-QSAR, docking, and molecular dynamics simulations studies. *Journal of Biomolecular Structure & Dynamics* 36, 1529-1549.
Darden, T., York, D., and Pedersen, L. (1993). Particle mesh Ewald: An N · log(N) method for Ewald sums in large systems. *The Journal of Chemical Physics* 98, 10089-10092.
Feller, S. E., Zhang, Y. H., Pastor, R. W., and Brooks, B. R. (1995). Constant pressure

molecular dynamics simulation: The Langevin piston method. *Journal of Chemical Physics* 103, 4613-4621.

Frisch, M. J., Trucks, G. W., Schlegel, H. B., Scuseria, G. E., Robb, M. A., Cheeseman, J. R., Scalmani, G., Barone, V., Petersson, G. A., Nakatsuji, H., Li, X., Caricato, M., Marenich, A. V., Bloino, J., Janesko, B. G., Gomperts, R., Mennucci, B., Hratchian, H. P., Ortiz, J. V., Izmaylov, A. F., Sonnenberg, J. L., Williams, Ding, F., Lipparini, F., Egidi, F., Goings, J., Peng, B., Petrone, A., Henderson, T., Ranasinghe, D., Zakrzewski, V. G., Gao, J., Rega, N., Zheng, G., Liang, W., Hada, M., Ehara, M., Toyota, K., Fukuda, R., Hasegawa, J., Ishida, M., Nakajima, T., Honda, Y., Kitao, O., Nakai, H., Vreven, T., Throssell, K., Montgomery Jr., J. A., Peralta, J. E., Ogliaro, F., Bearpark, M. J., Heyd, J. J., Brothers, E. N., Kudin, K. N., Staroverov, V. N., Keith, T. A., Kobayashi, R., Normand, J., Raghavachari, K., Rendell, A. P., Burant, J. C., Iyengar, S. S., Tomasi, J., Cossi, M., Millam, J. M., Klene, M., Adamo, C., Cammi, R., Ochterski, J. W., Martin, R. L., Morokuma, K., Farkas, O., Foresman, J. B., and Fox, D. J. (2016). Gaussian 16 Rev. C.01. (Wallingford, CT).

Gilson, M. K., and Zhou, H. X. (2007). Calculation of protein-ligand binding affinities. *Annual Review of Biophysics and Biomolecular Structure* 36, 21-42.

Gordon, J. C., Myers, J. B., Folta, T., Shoja, V., Heath, L. S., and Onufriev, A. (2005). H++: a server for estimating pK(a)s and adding missing hydrogens to macromolecules. *Nucleic Acids Research* 33, W368-W371.

Izadi, S., Anandakrishnan, R., and Onufriev, A. V. (2014). Building water models: A different approach. *Journal of Physical Chemistry Letters* 5, 3863-3871.

King, E., Aitchison, E., Li, H., and Luo, R. (2021). Recent developments in free energy calculations for drug discovery. *Frontiers in Molecular Biosciences* 8, 24.

Lee, F. S., Chu, Z. T., Bolger, M. B., and Warshel, A. (1992). Calculations of antibody-antigen interactions: microscopic and semi-microscopic evaluation of the free energies of binding of phosphorylcholine analogs to McPC603. *Protein Engineering* 5, 215-228.

Lee, M. S., Salsbury, F. R., and Olson, M. A. (2004). An efficient hybrid explicit/implicit solvent method for biomolecular simulations. *Journal of Computational Chemistry* 25, 1967-1978.

Li, Y. B., Pei, J. F., and Lai, L. H. (2021). Structure-based de novo drug design using 3D deep generative models. *Chemical Science* 12, 13664-13675.

Martyna, G. J., Tobias, D. J., and Klein, M. L. (1994). Constant pressure molecular

dynamics algorithms. *Journal of Chemical Physics* 101**,** 4177-4189.

Morris, G. M., Huey, R., Lindstrom, W., Sanner, M. F., Belew, R. K., Goodsell, D. S., and Olson, A. J. (2009). AutoDock4 and AutoDockTools4: Automated docking with selective receptor flexibility. *Journal of Computational Chemistry* 30**,** 2785-2791.

Myers, J., Grothaus, G., Narayanan, S., and Onufriev, A. (2006). A simple clustering algorithm can be accurate enough for use in calculations of pKs in macromolecules. *Proteins-Structure Function and Bioinformatics* 63**,** 928-938.

Naim, M., Bhat, S., Rankin, K. N., Dennis, S., Chowdhury, S. F., Siddiqi, I., Drabik, P., Sulea, T., Bayly, C. I., Jakalian, A., and Purisima, E. O. (2007). Solvated interaction energy (SIE) for scoring protein-ligand binding affinities. 1. Exploring the parameter space. *Journal of Chemical Information and Modeling* 47**,** 122-133.

Perdih, A., Bren, U., and Solmajer, T. (2009). Binding free energy calculations of N-sulphonyl-glutamic acid inhibitors of MurD ligase. *Journal of Molecular Modeling* 15**,** 983-996.

Roe, D. R., and Cheatham, T. E. (2013). PTRAJ and CPPTRAJ: Software for processing and analysis of molecular dynamics trajectory data. *Journal of Chemical Theory and Computation* 9**,** 3084-3095.

Roe, D. R., and Cheatham, T. E. (2018). Parallelization of CPPTRAJ enables large scale analysis of molecular dynamics trajectory data. *Journal of Computational Chemistry* 39**,** 2110-2117.

Ryckaert, J.-P., Ciccotti, G., and Berendsen, H. J. C. (1977). Numerical integration of the cartesian equations of motion of a system with constraints: molecular dynamics of n-alkanes. *Journal of Computational Physics* 23**,** 327-341.

Sanner, M. F. (1999). Python: A programming language for software integration and development. *Journal of Molecular Graphics & Modelling* 17**,** 57-61.

Shi, M., Chen, T., Wei, S., Zhao, C., Zhang, X., Li, X., Tang, X., Liu, Y., Yang, Z., and Chen, L. (2022). Molecular docking, molecular dynamics simulations, and free energy calculation insights into the binding mechanism between VS-4718 and focal adhesion kinase. *ACS Omega* 7**,** 32442-32456.

Shi, M., Wang, F., Lu, Z., Yin, Y., Zheng, X., Wang, D., Cai, X., Jing, M., Wang, J., Chen, J., Jiang, X., Yu, W., and Li, X. (2024). Elucidating the linagliptin and fibroblast activation protein binding mechanism through molecular dynamics and binding free energy analysis. *iScience* 27**,** 111368.

Shi, M., Wang, L., Li, P., Liu, J., Chen, L., and Xu, D. (2021). Dasatinib-SIK2 binding elucidated by homology modeling, molecular docking, and dynamics simulations. *ACS Omega* 6, 11025-11038.

Shirvani, P., and Fassihi, A. (2022). In silico design of novel FAK inhibitors using integrated molecular docking, 3D-QSAR and molecular dynamics simulation studies. *Journal of Biomolecular Structure & Dynamics* 40, 5965-5982.

Srinivasan, J., Cheatham, T. E., Cieplak, P., Kollman, P. A., and Case, D. A. (1998). Continuum solvent studies of the stability of DNA, RNA, and phosphoramidate - DNA helices. *Journal of the American Chemical Society* 120, 9401-9409.

Srinivasan, J., Trevathan, M. W., Beroza, P., and Case, D. A. (1999). Application of a pairwise generalized Born model to proteins and nucleic acids: inclusion of salt effects. *Theoretical Chemistry Accounts* 101, 426-434.

Still, W. C., Tempczyk, A., Hawley, R. C., and Hendrickson, T. (1990). Semianalytical treatment of solvation for molecular mechanics and dynamics. *Journal of the American Chemical Society* 112, 6127-6129.

Tian, C., Kasavajhala, K., Belfon, K. A. A., Raguette, L., Huang, H., Migues, A. N., Bickel, J., Wang, Y. Z., Pincay, J., Wu, Q., and Simmerling, C. (2020). ff19SB: Amino-acid-specific protein backbone parameters trained against quantum mechanics energy surfaces in solution. *Journal of Chemical Theory and Computation* 16, 528-552.

Wang, J. M., Wolf, R. M., Caldwell, J. W., Kollman, P. A., and Case, D. A. (2004). Development and testing of a general amber force field. *Journal of Computational Chemistry* 25, 1157-1174.

Weiser, J., Shenkin, P. S., and Still, W. C. (1999). Approximate atomic surfaces from linear combinations of pairwise overlaps (LCPO). *Journal of Computational Chemistry* 20, 217-230.

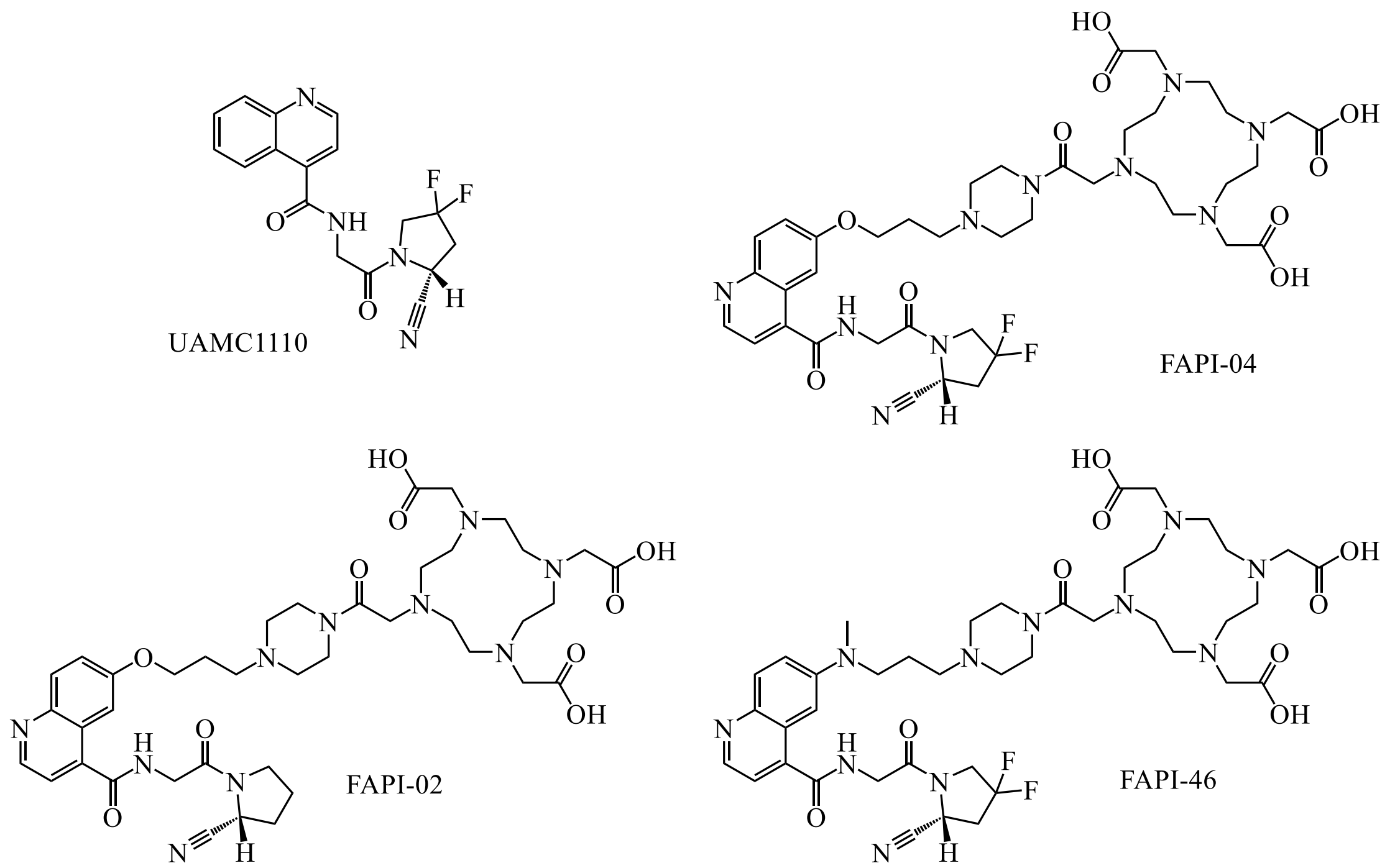

Figure S1 Structures for main FAP inhibitors

Ligand878

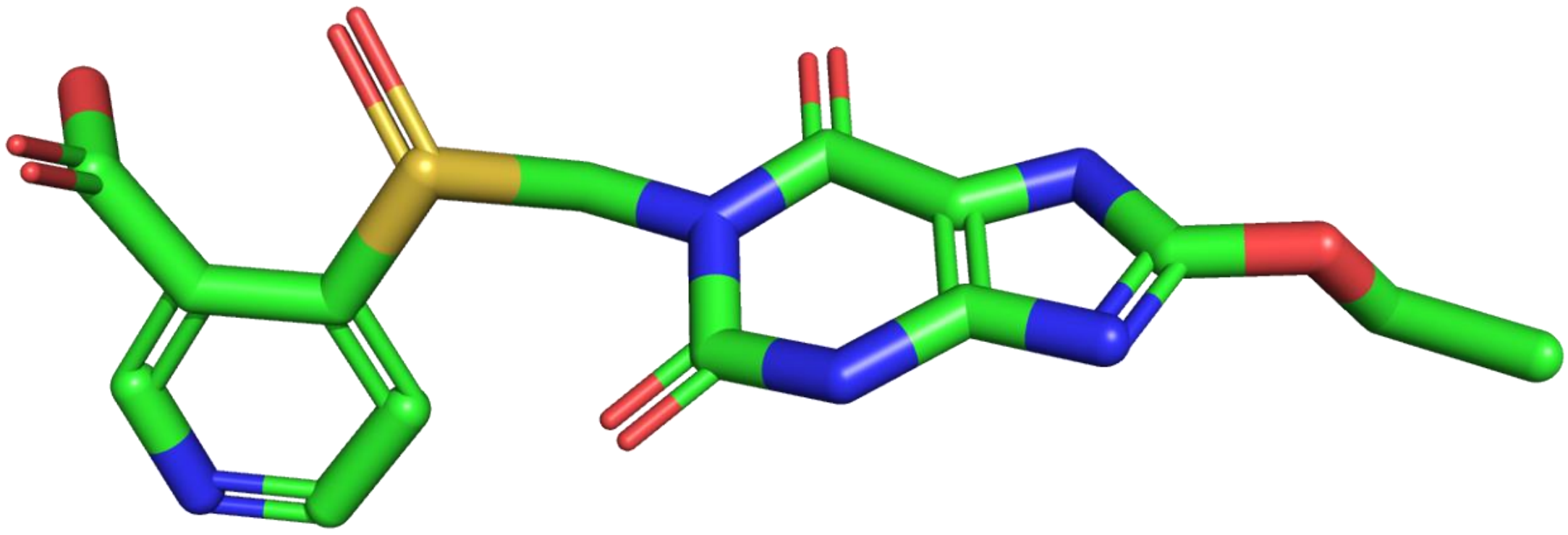

Figure S2 Structure of Ligand878

The structure of Ligand878 was generated using generative AI from DeepLigBuilder in PharmaMind v.3.12.

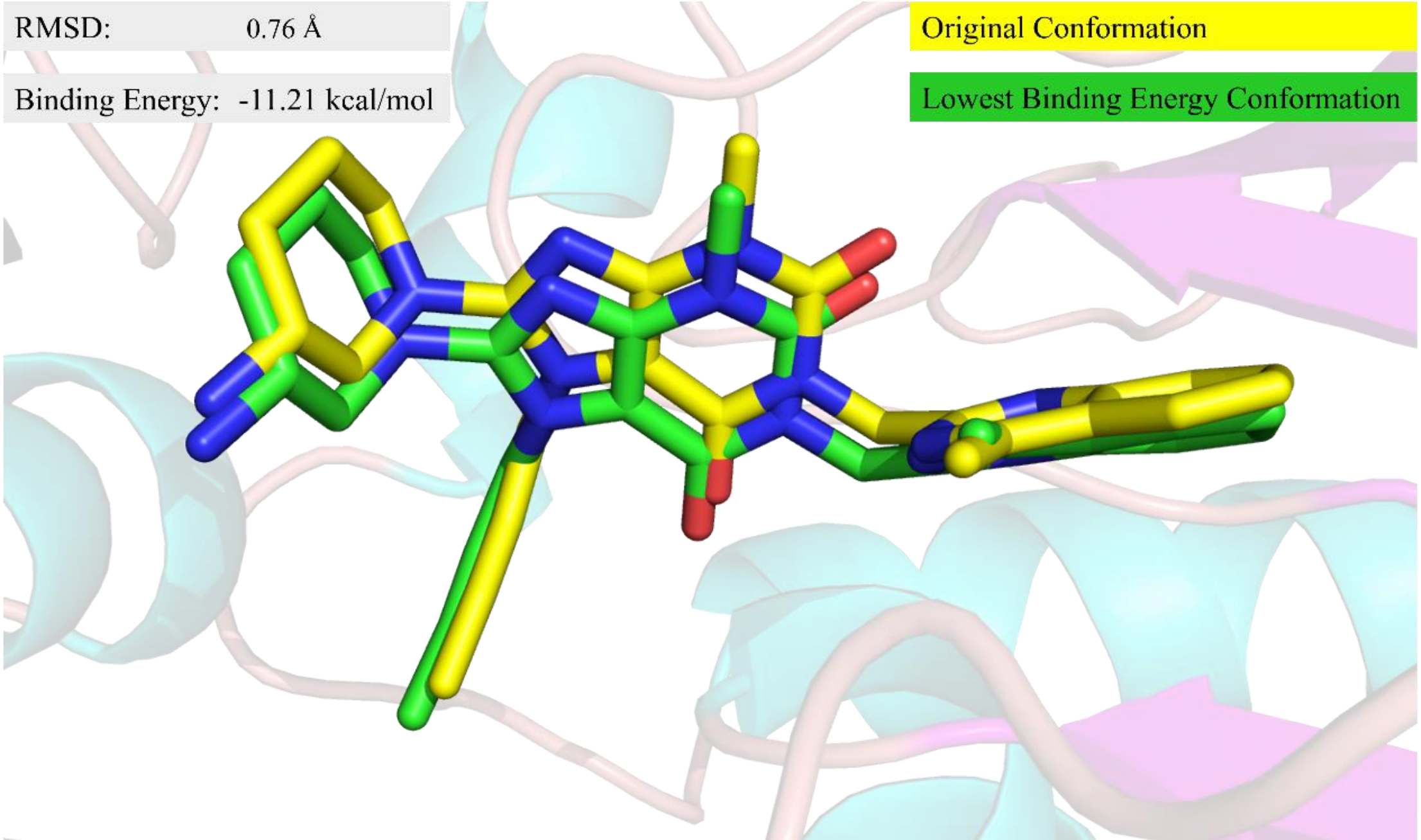

Figure S3 Docking results for original linagliptin structure with FAP

For clarity, the ligand is plotted using stick style, while cartoon style for human FAP.

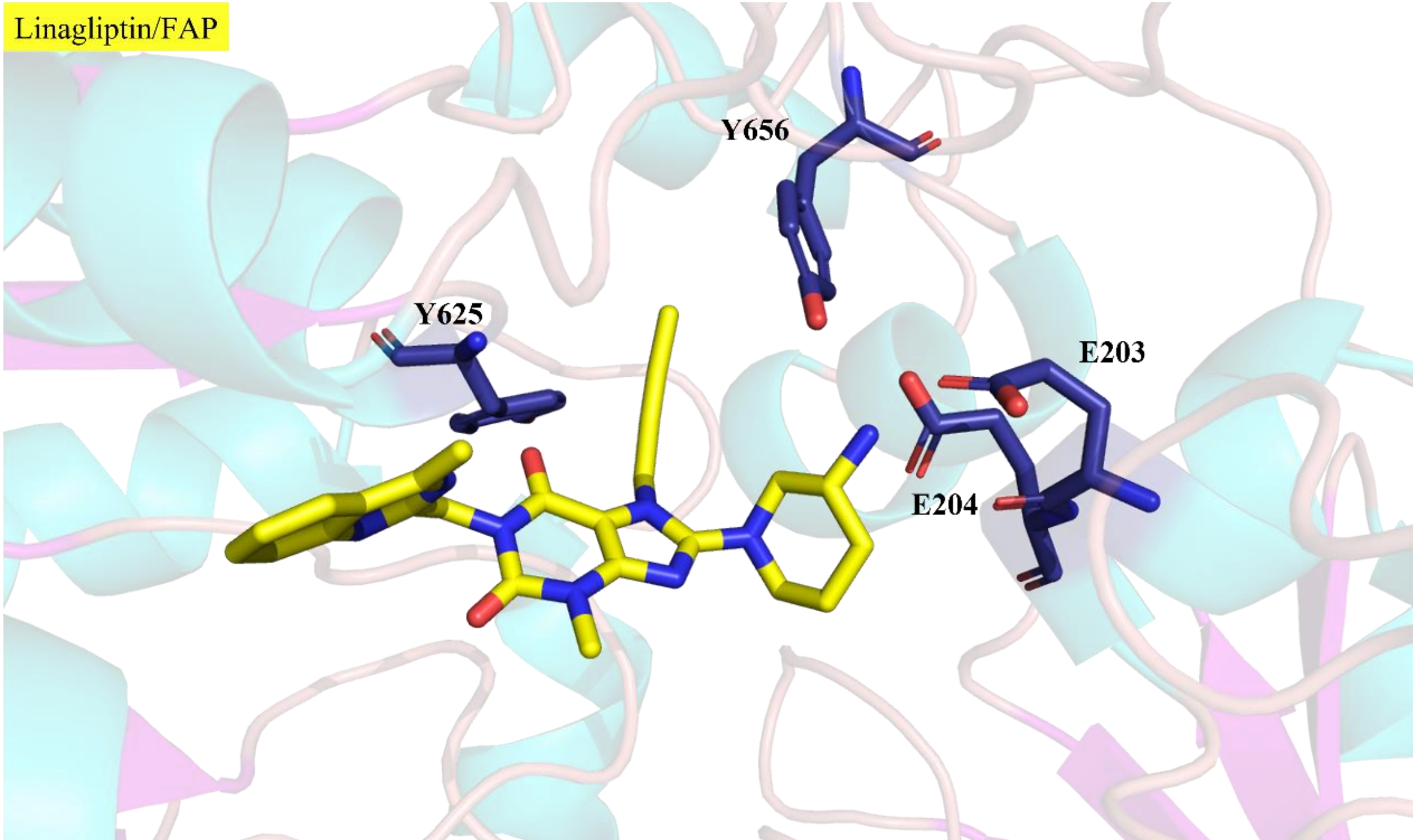

Figure S4 Initial linagliptin/FAP complex system

The ligand (stick style) is shown in the FAP binding pocket (cartoon style) with key interacting residues (stick style) labeled: E203 (Glu203), E204 (Glu204), Y625 (Tyr625), and Y656 (Tyr656).

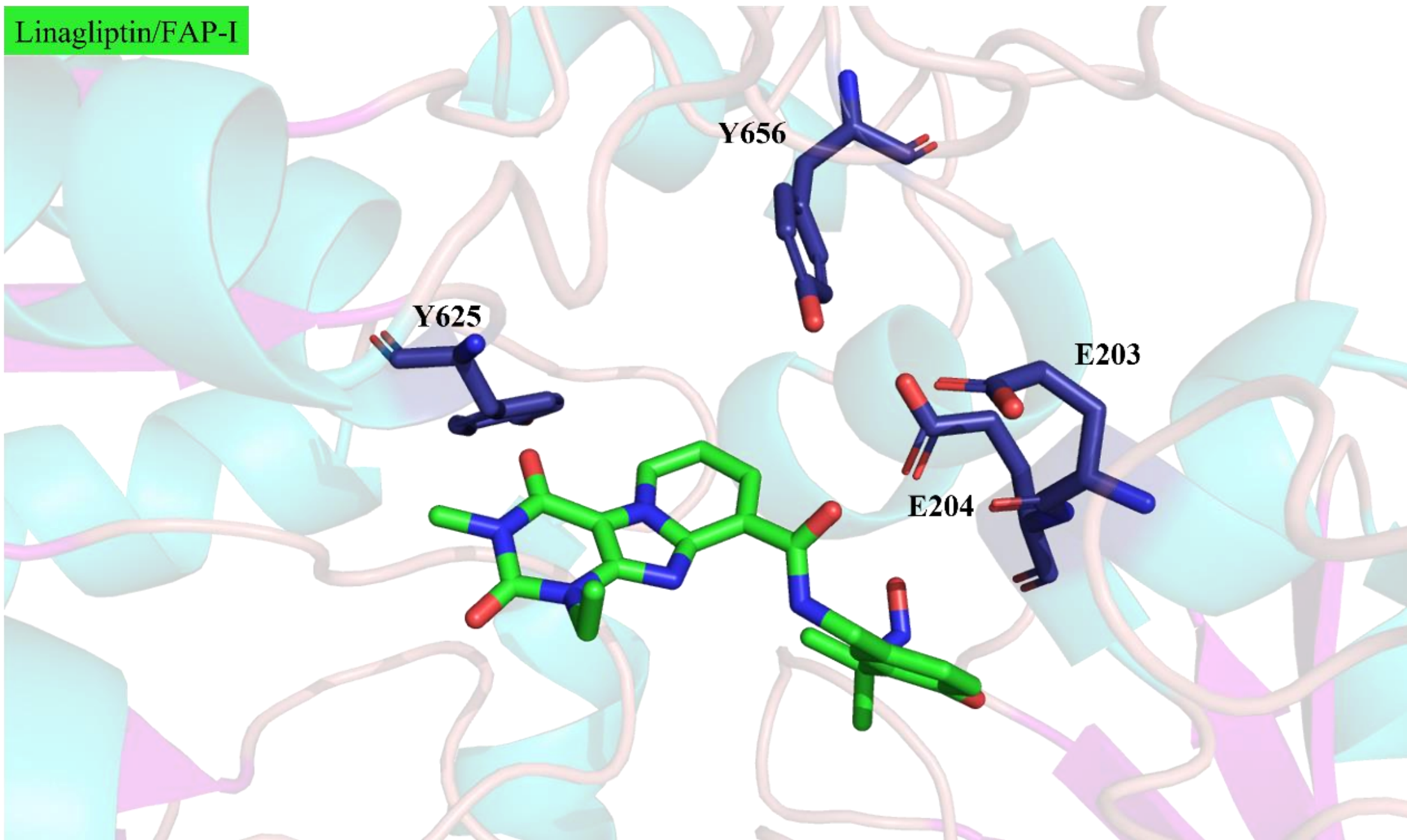

Figure S5 Initial ligand/FAP-I complex system

The ligand (stick style) is shown in the FAP binding pocket (cartoon style) with key interacting residues (stick style) labeled: E203 (Glu203), E204 (Glu204), Y625 (Tyr625), and Y656 (Tyr656).

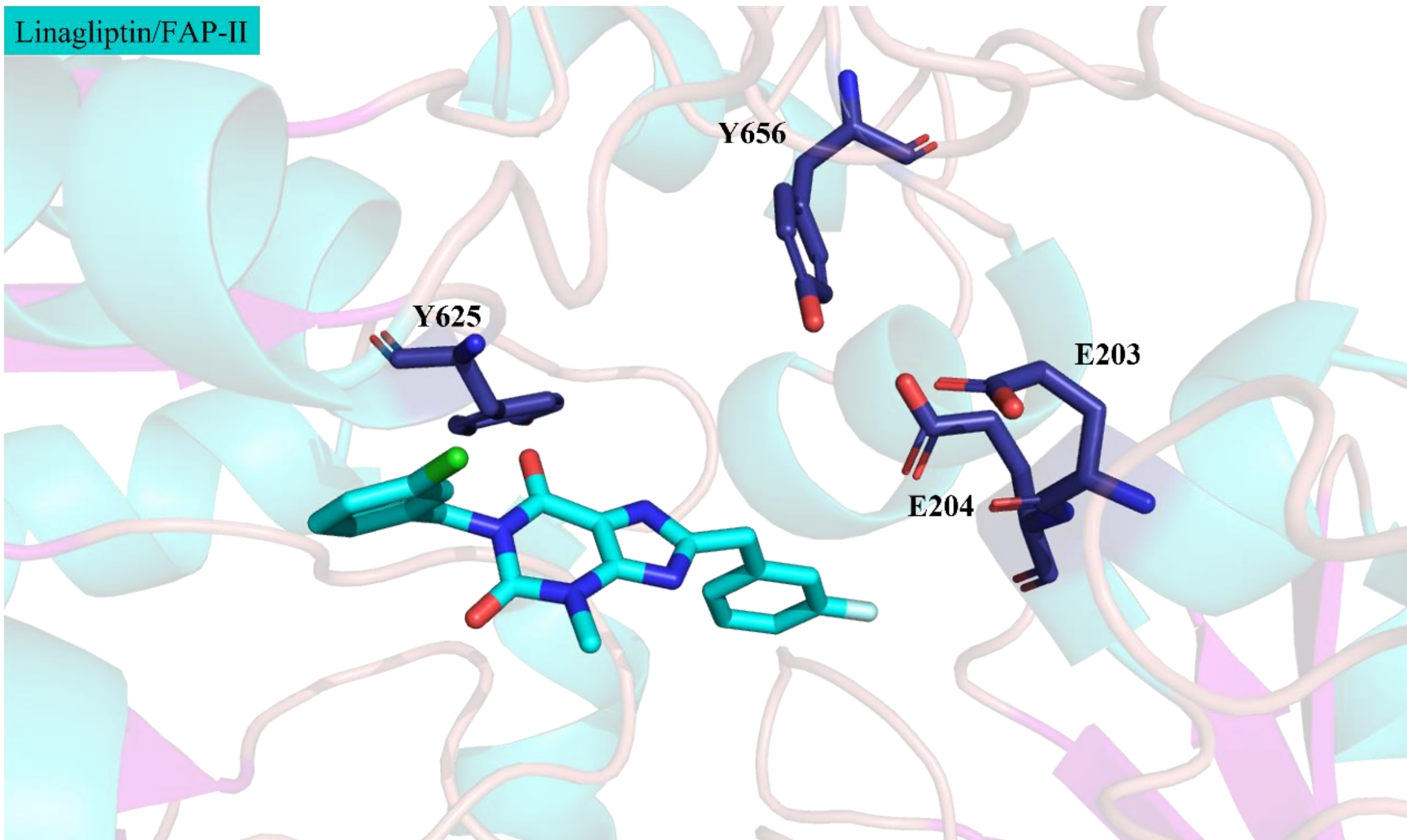

Figure S6 Initial ligand/FAP-II complex system

The ligand (stick style) is shown in the FAP binding pocket (cartoon style) with key interacting residues (stick style) labeled: E203 (Glu203), E204 (Glu204), Y625 (Tyr625), and Y656 (Tyr656).

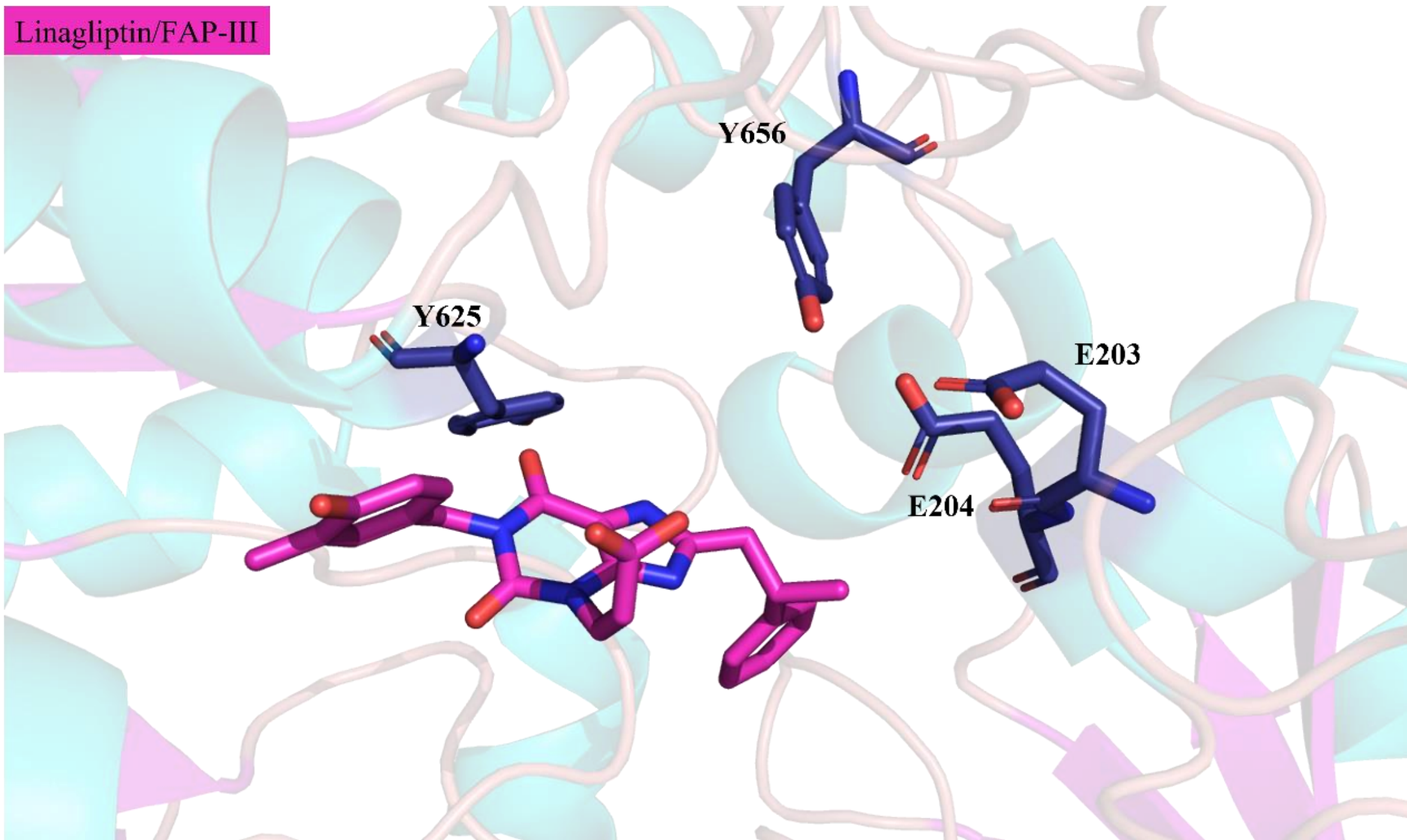

Figure S7 Initial ligand/FAP-III complex system

The ligand (stick style) is shown in the FAP binding pocket (cartoon style) with key interacting residues (stick style) labeled: E203 (Glu203), E204 (Glu204), Y625 (Tyr625), and Y656 (Tyr656).

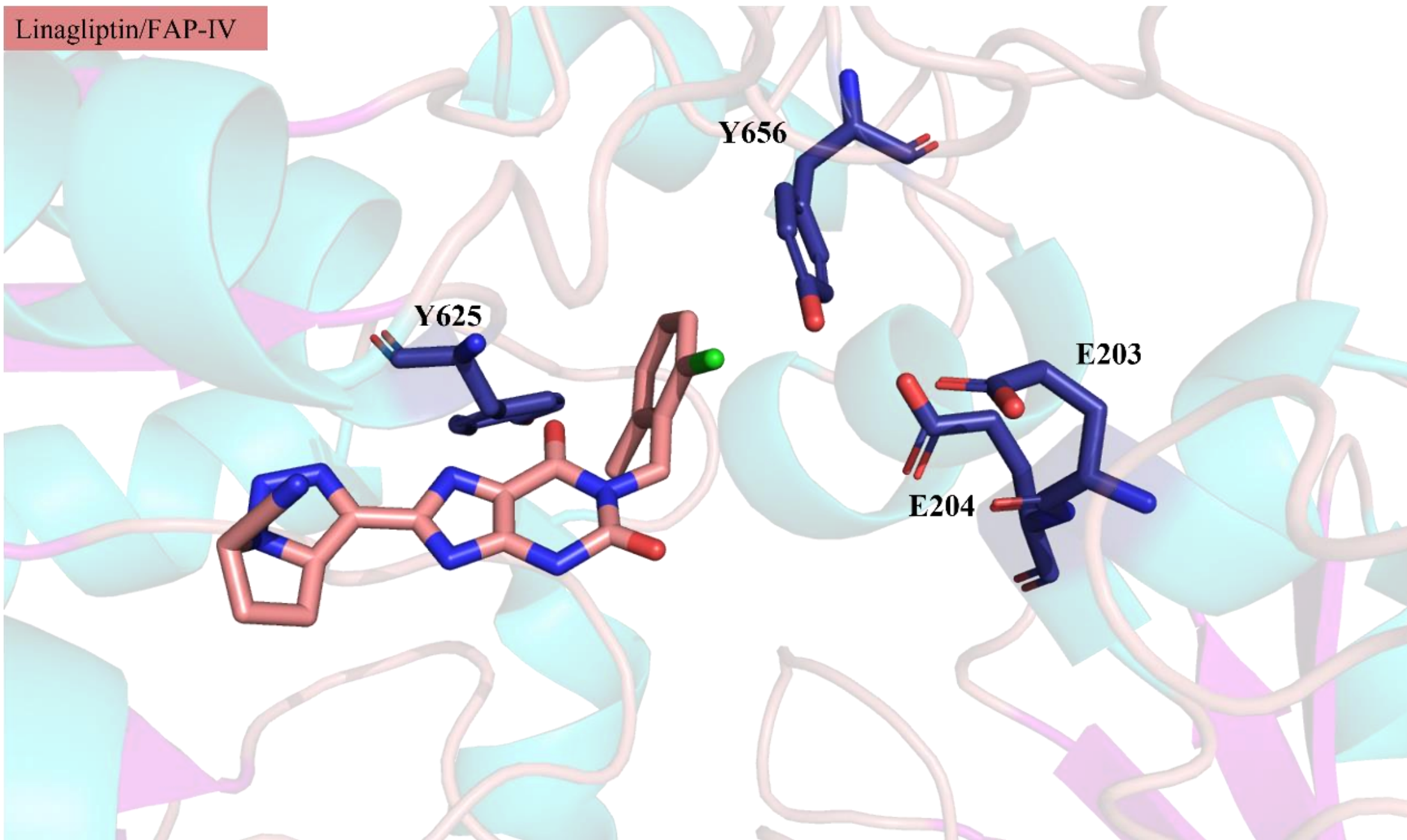

Figure S8 Initial ligand/FAP-IV complex system

The ligand (stick style) is shown in the FAP binding pocket (cartoon style) with key interacting residues (stick style) labeled: E203 (Glu203), E204 (Glu204), Y625 (Tyr625), and Y656 (Tyr656).

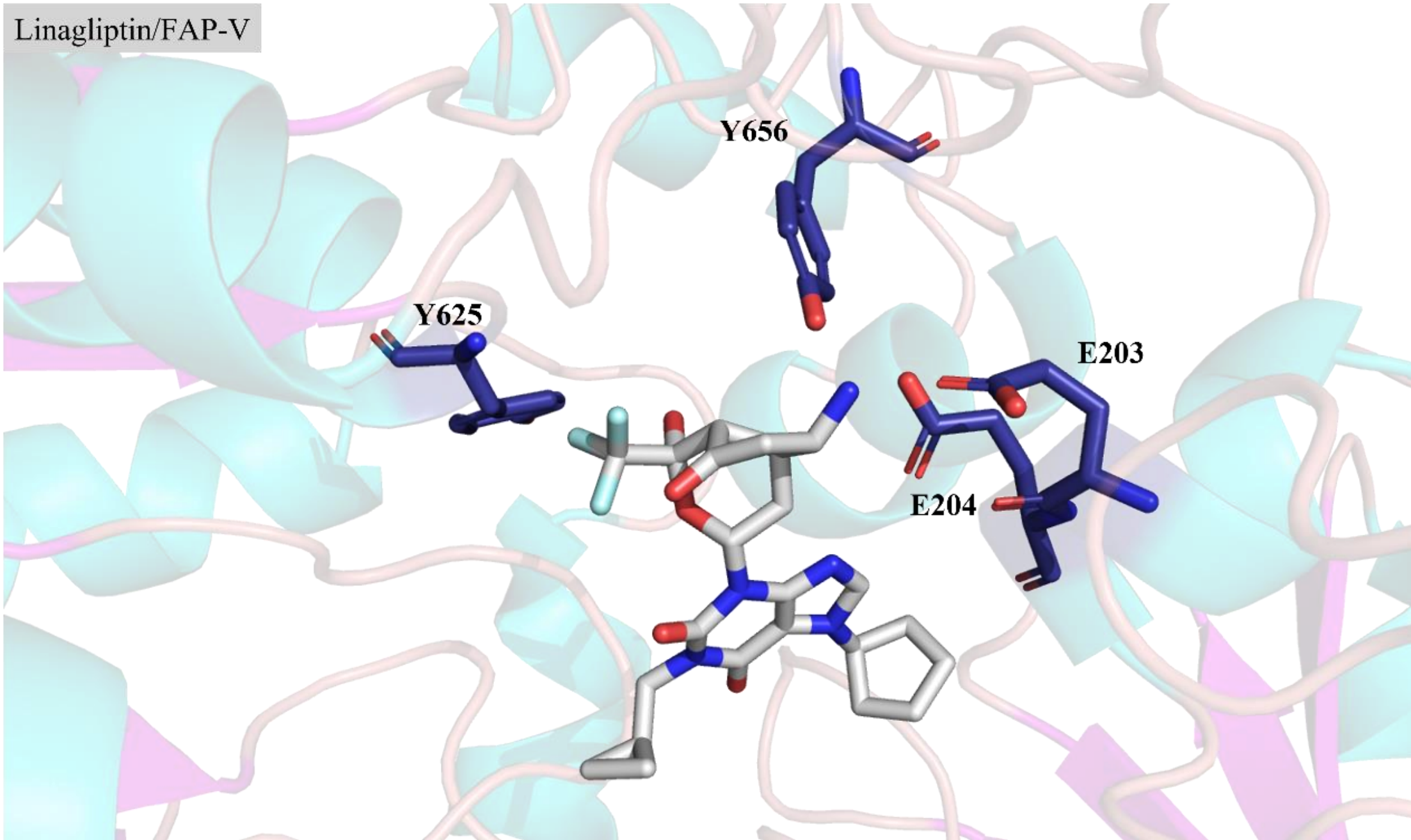

Figure S9 Initial ligand/FAP-V complex system

The ligand (stick style) is shown in the FAP binding pocket (cartoon style) with key interacting residues (stick style) labeled: E203 (Glu203), E204 (Glu204), Y625 (Tyr625), and Y656 (Tyr656).

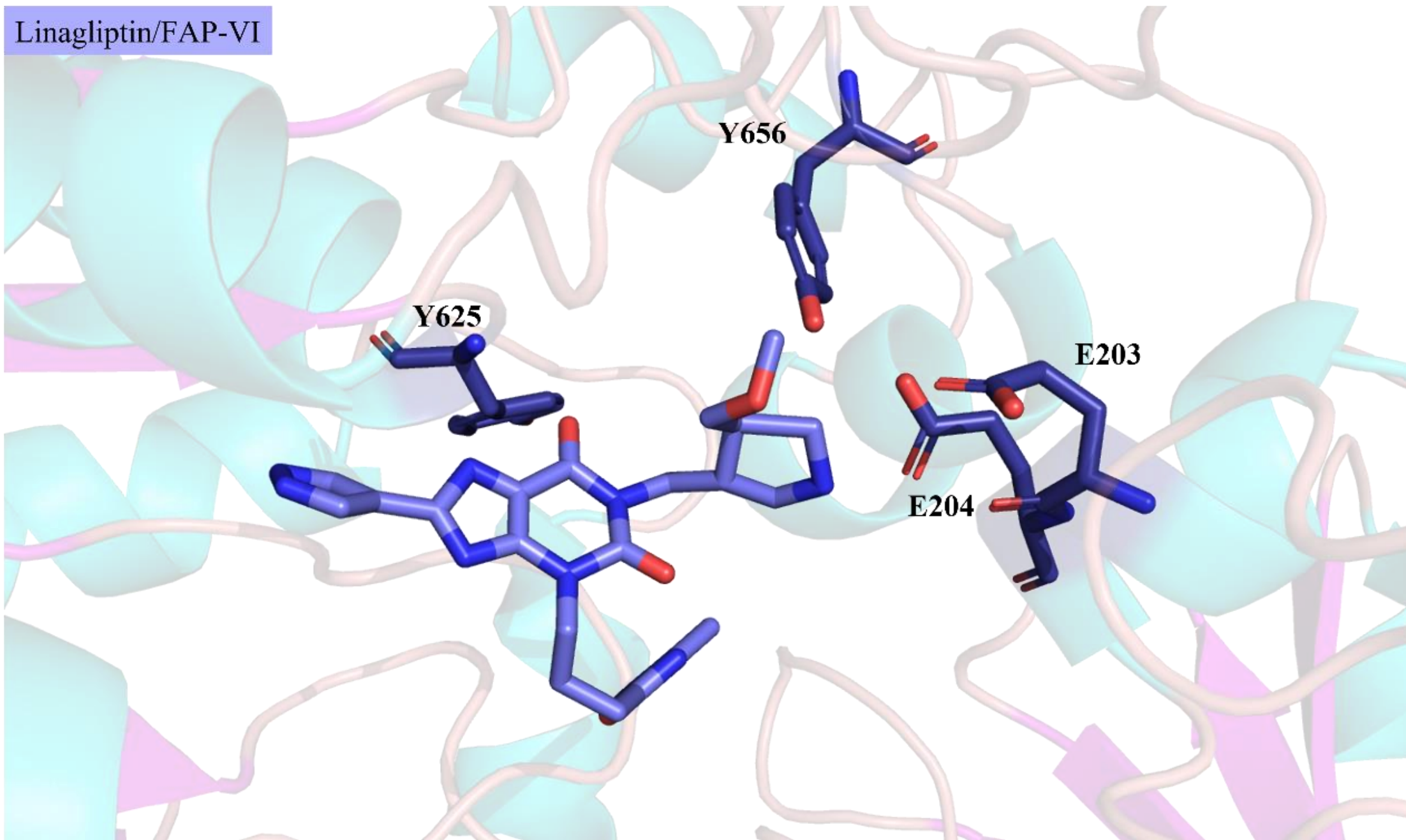

Figure S10 Initial ligand/FAP-VI complex system

The ligand (stick style) is shown in the FAP binding pocket (cartoon style) with key interacting residues (stick style) labeled: E203 (Glu203), E204 (Glu204), Y625 (Tyr625), and Y656 (Tyr656).

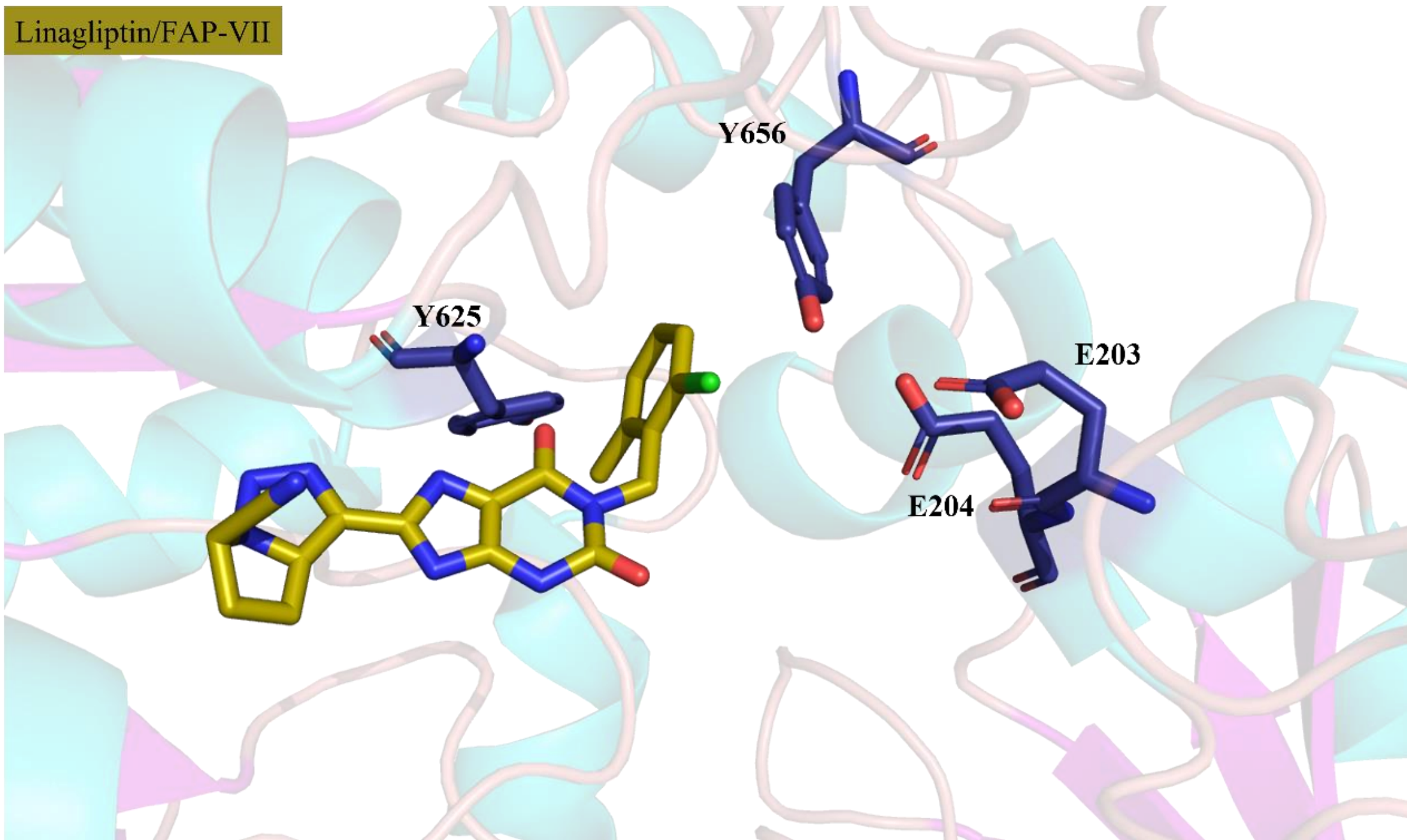

Figure S11 Initial ligand/FAP-VII complex system

The ligand (stick style) is shown in the FAP binding pocket (cartoon style) with key interacting residues (stick style) labeled: E203 (Glu203), E204 (Glu204), Y625 (Tyr625), and Y656 (Tyr656).

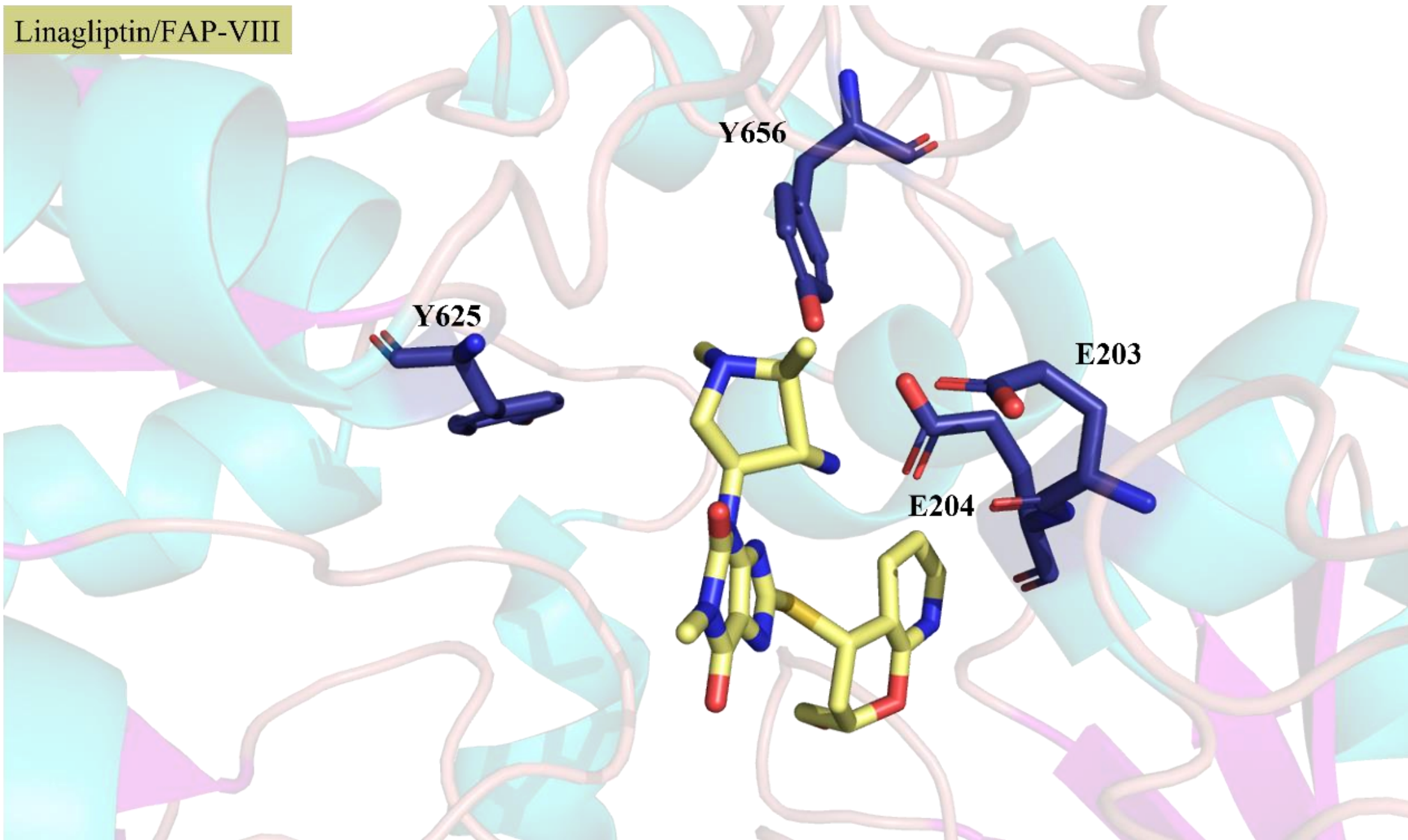

Figure S12 Initial ligand/FAP-VIII complex system

The ligand (stick style) is shown in the FAP binding pocket (cartoon style) with key interacting residues (stick style) labeled: E203 (Glu203), E204 (Glu204), Y625 (Tyr625), and Y656 (Tyr656).

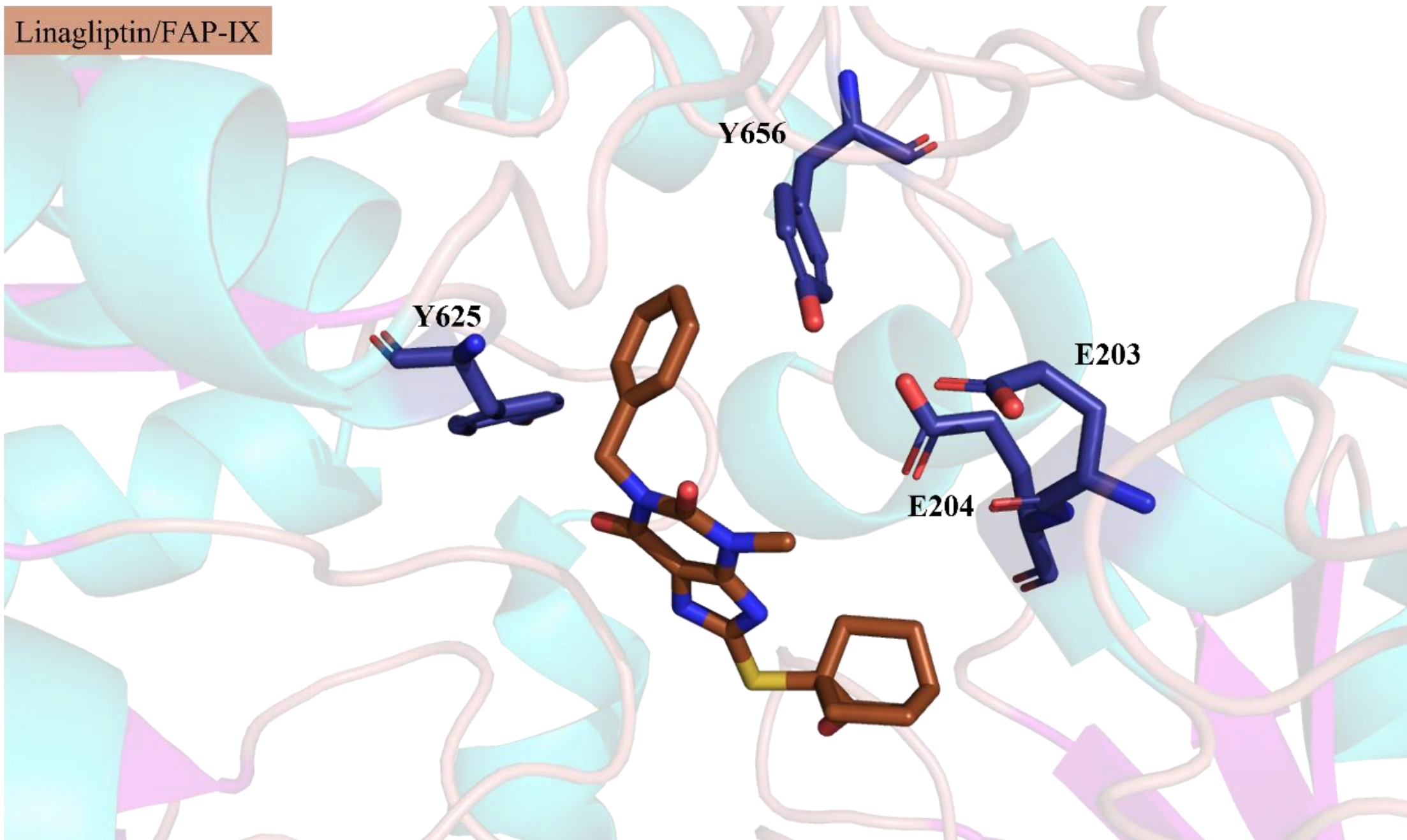

Figure S13 Initial ligand/FAP-IX complex system

The ligand (stick style) is shown in the FAP binding pocket (cartoon style) with key interacting residues (stick style) labeled: E203 (Glu203), E204 (Glu204), Y625 (Tyr625), and Y656 (Tyr656).

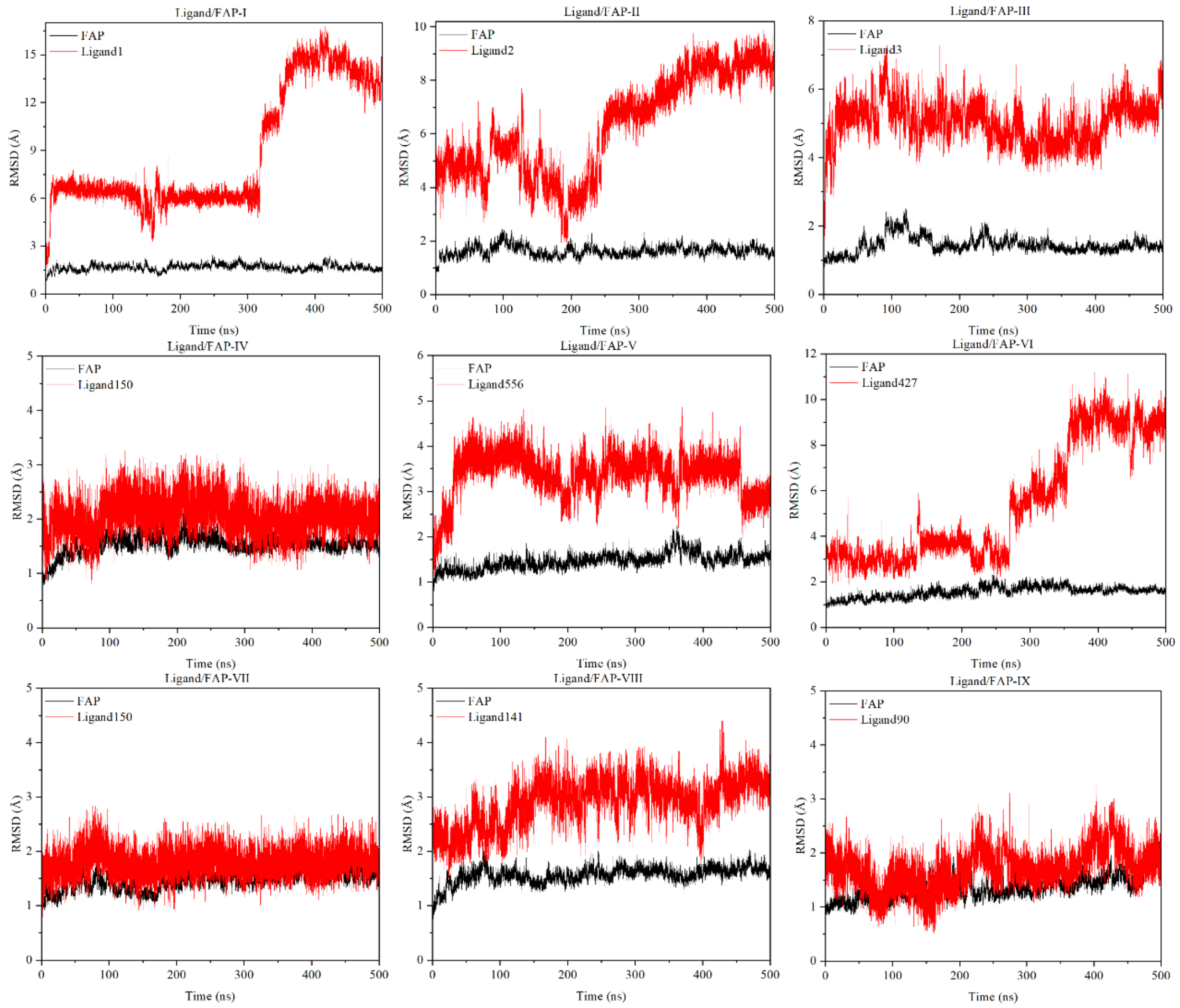

Figure S14 Root mean square deviation (RMSD) of the backbone heavy atoms in human FAP and non-hydrogen atoms of the ligands during the 500-ns MD simulations of the Ligand/FAP complex systems

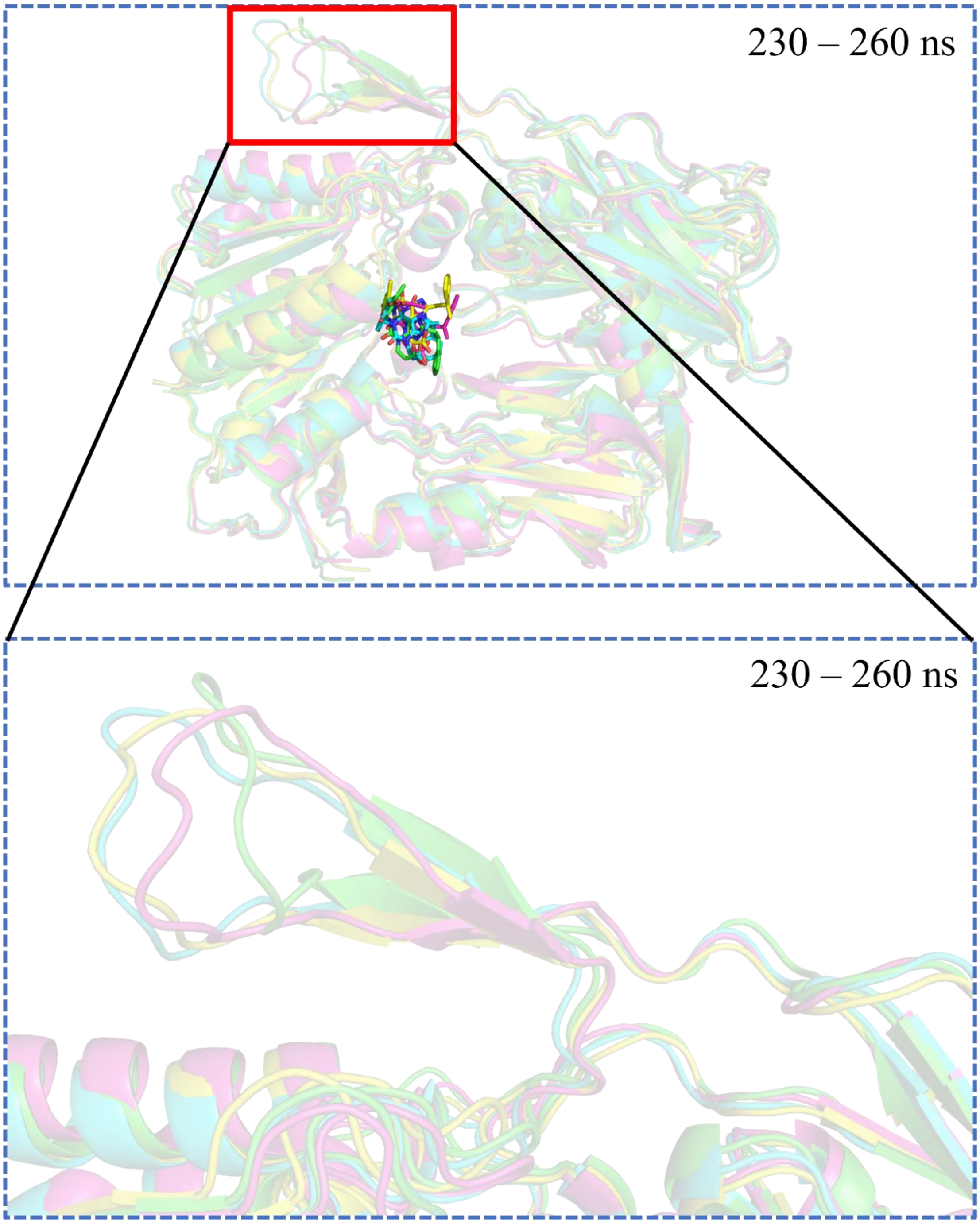

Figure S15 Snapshots of the Ligand/FAP-III complex during the MD simulation at 230-260 ns

For clarity, the water molecules have been removed. The ligand is plotted using stick style, while cartoon style for human FAP.

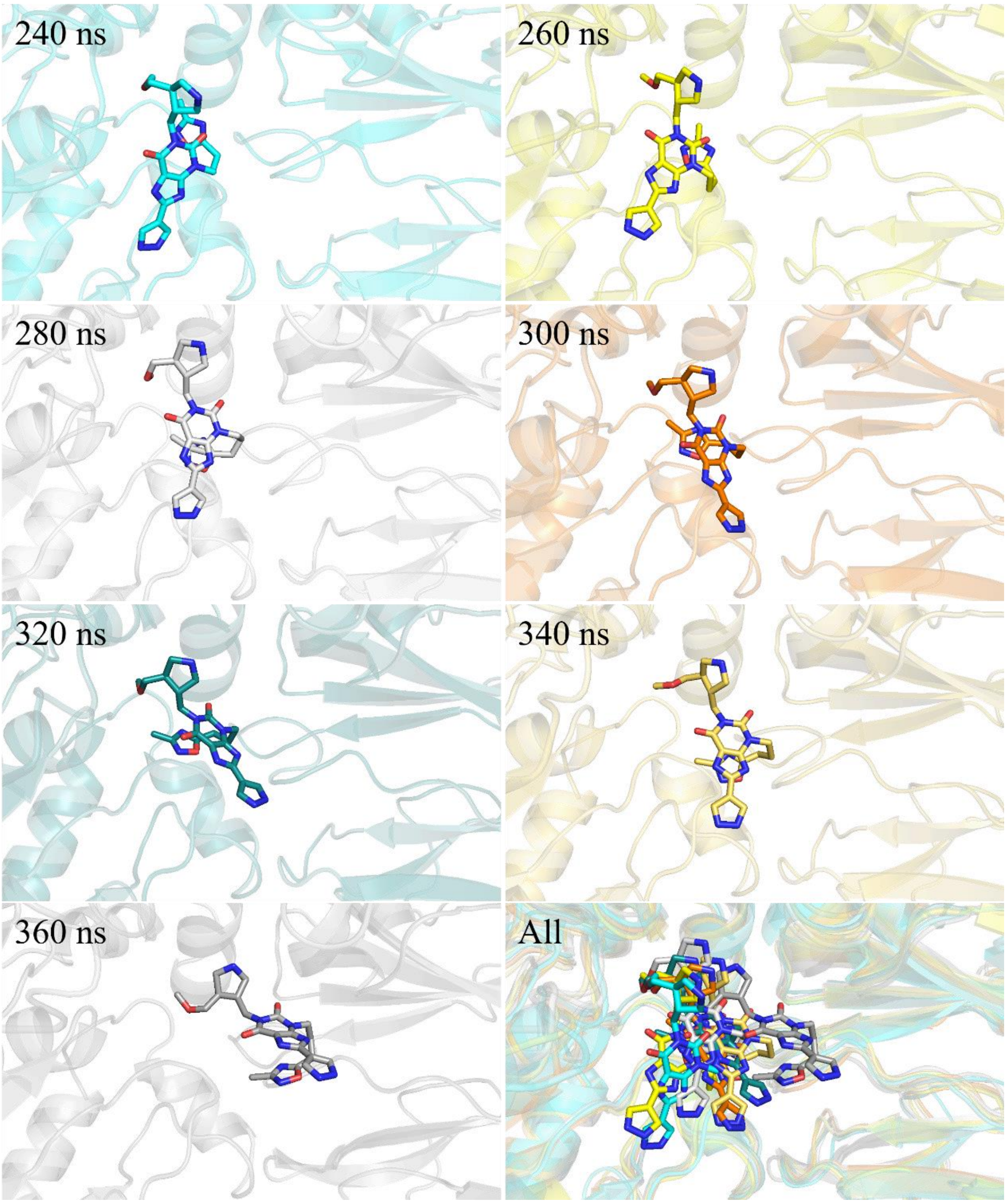

Figure S16 Snapshots of the Ligand/FAP-VI complex during the MD simulation at 240-360 ns

For clarity, the water molecules have been removed. The ligand is plotted using stick style, whereas cartoon style has been used for human FAP.

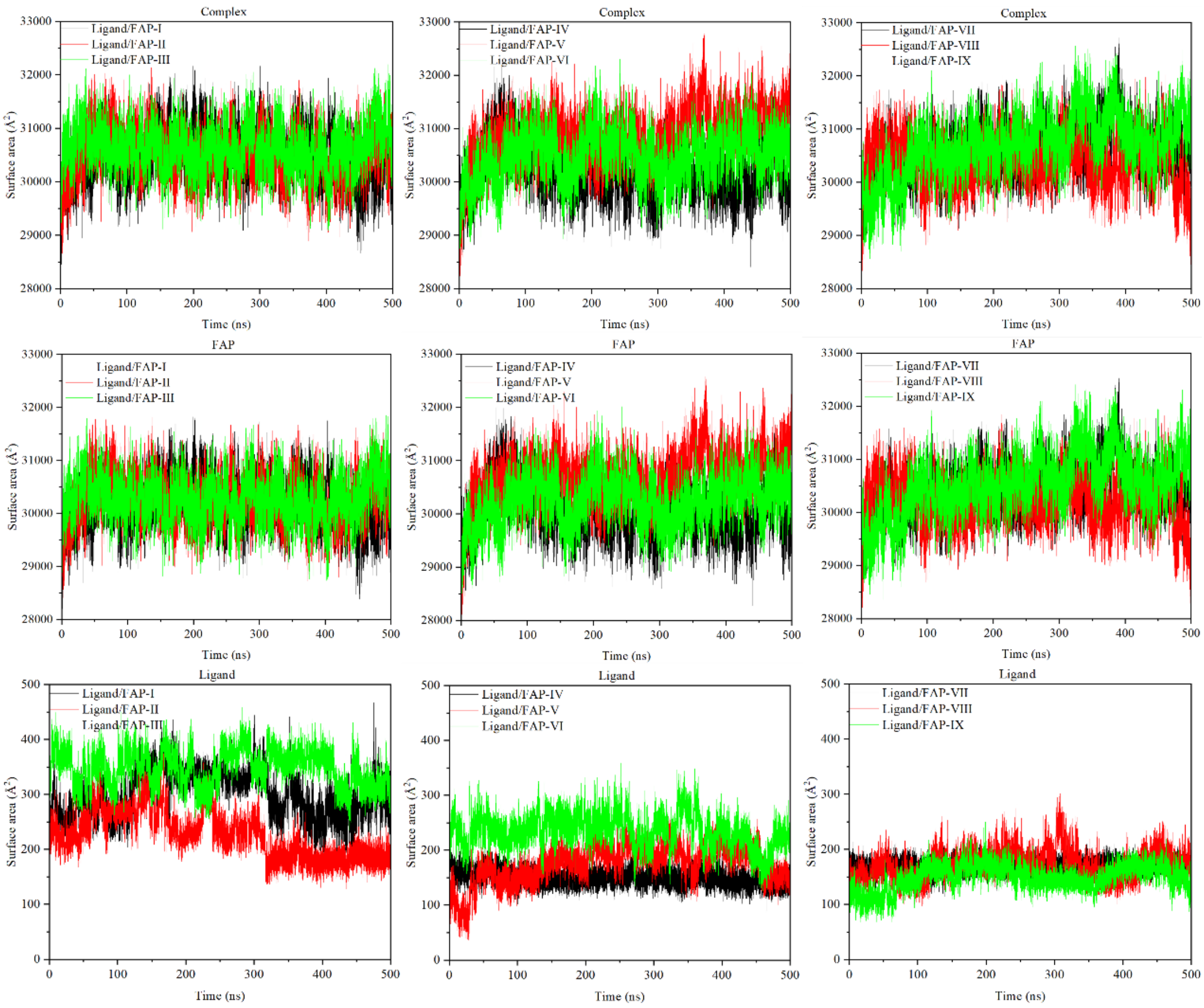

Figure S17 Surface area for ligand binding with FAP

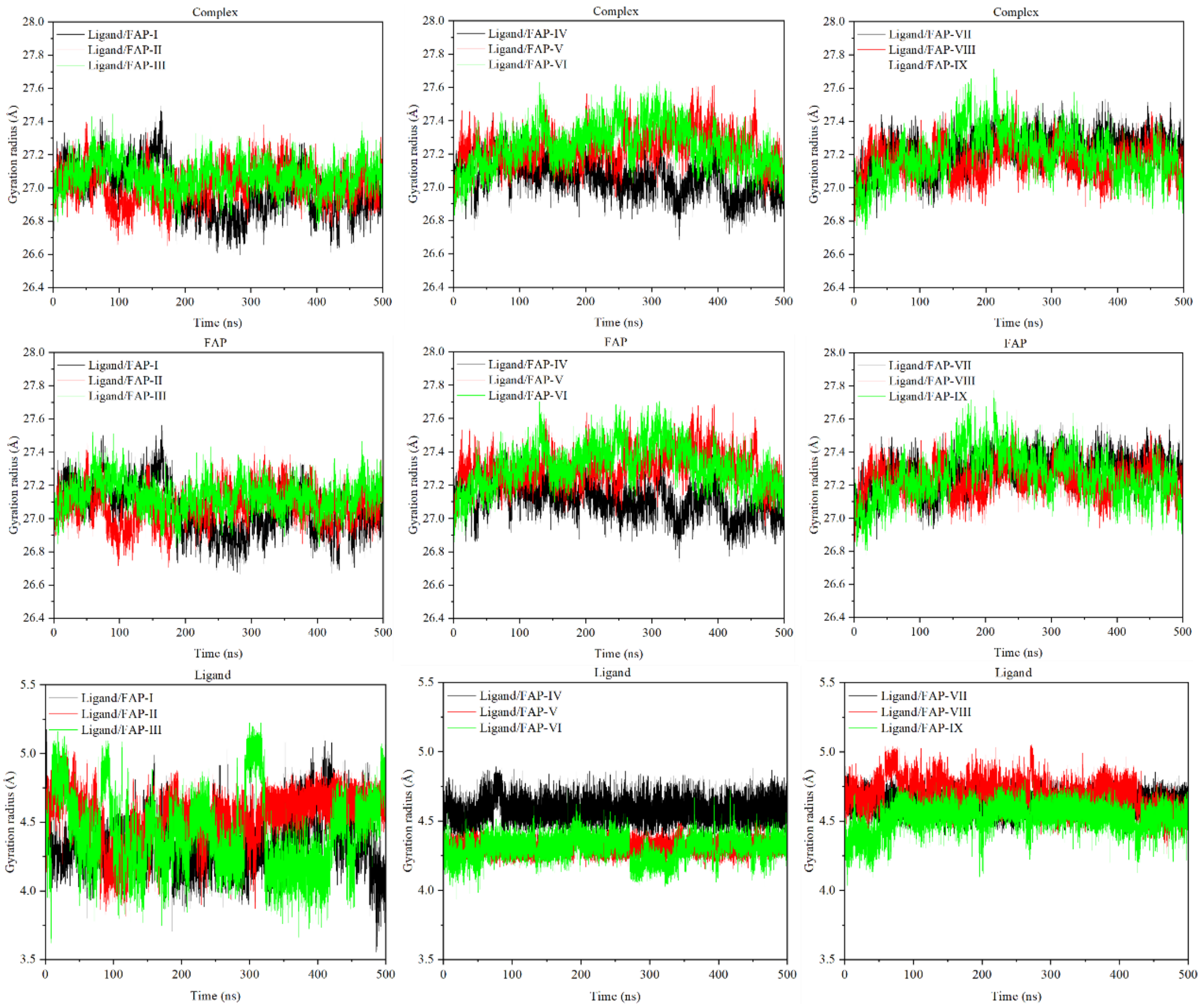

Figure S18 Gyration radius for ligand binding with FAP

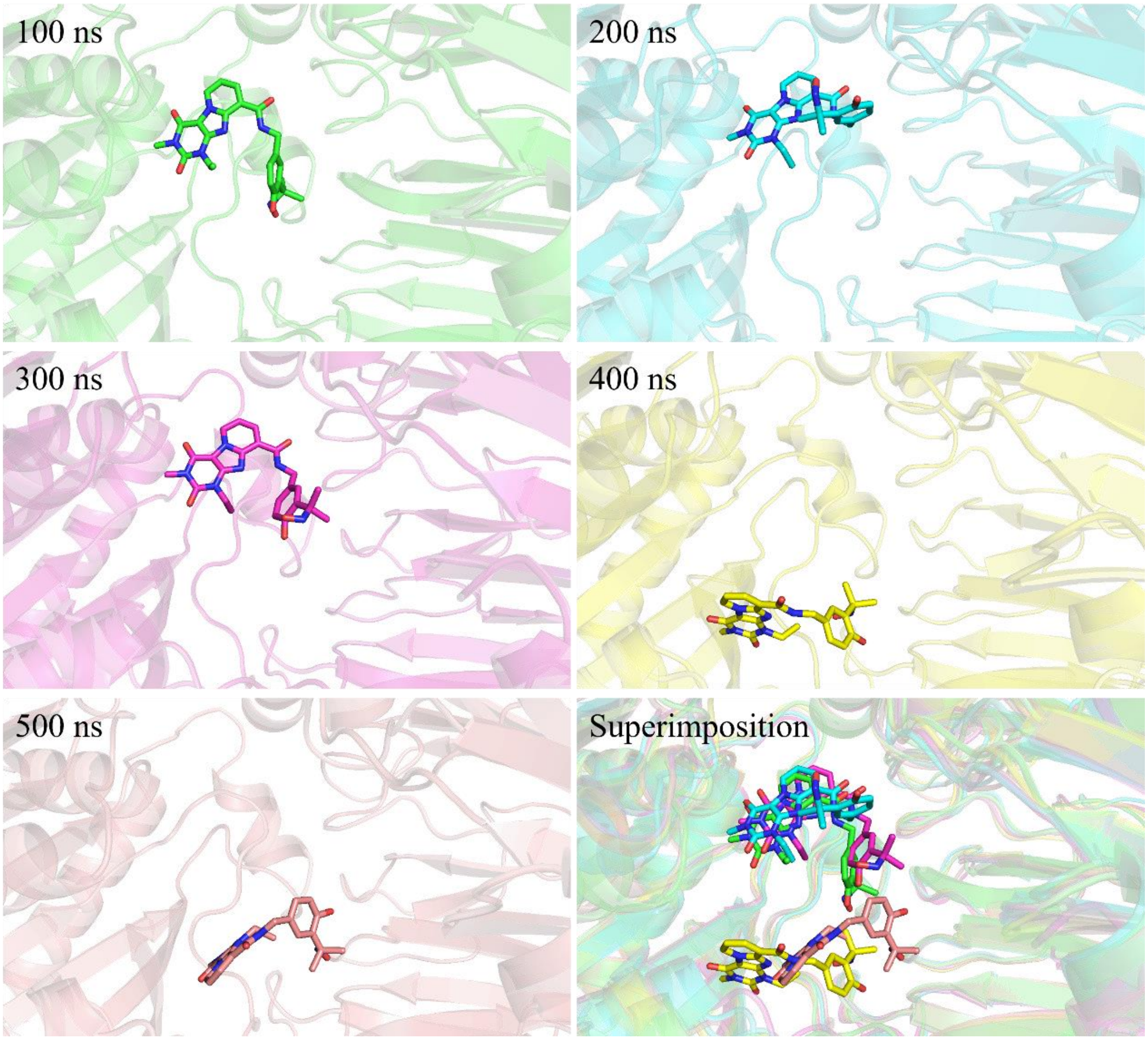

Figure S19 Snapshots of the Ligand/FAP-I complex during the MD simulation at 100, 200, 300, 400, and 500 ns

For clarity, the water molecules have been removed. The ligand is plotted using stick style, while human FAP is presented in cartoon style.

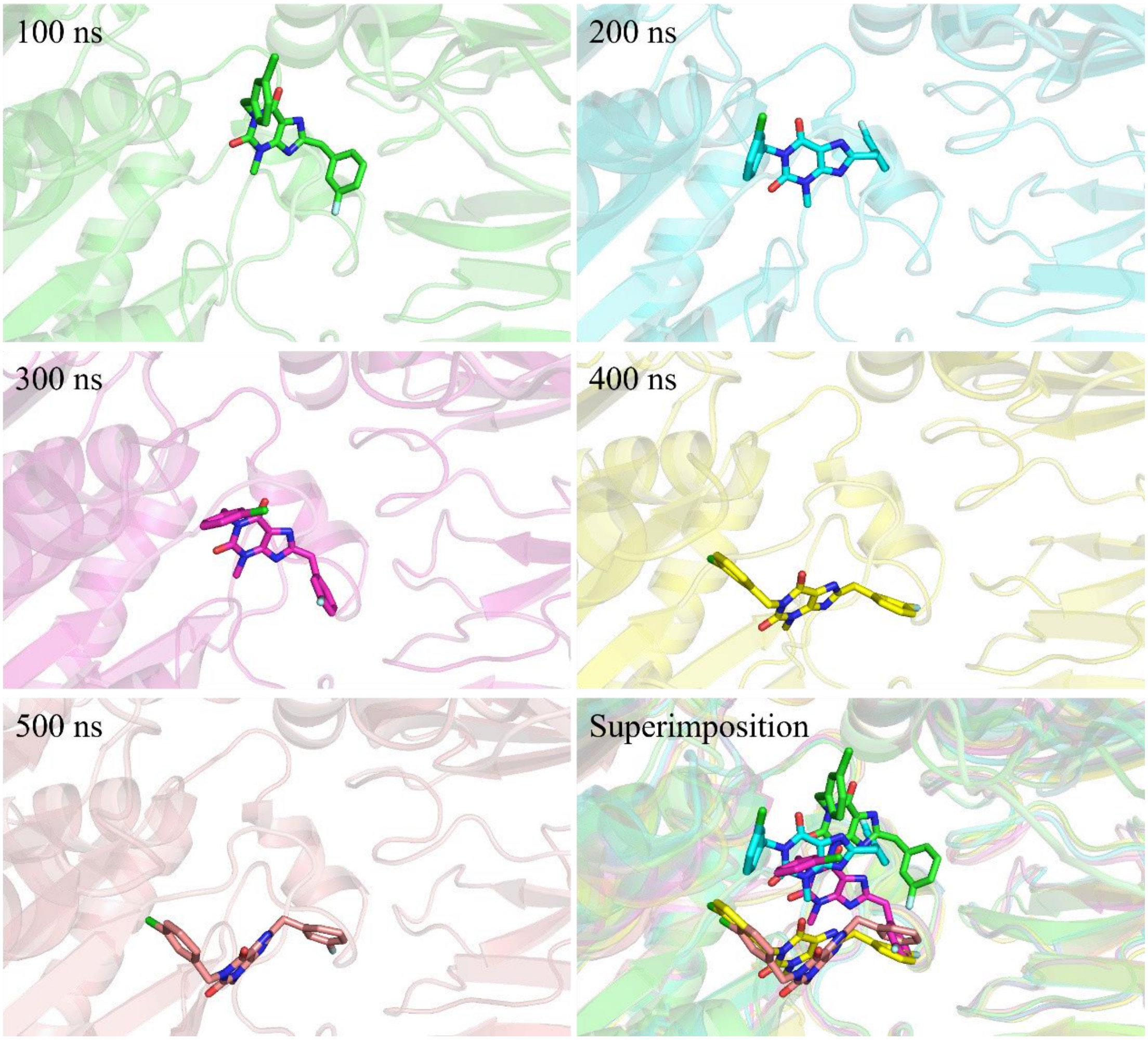

Figure S20 Snapshots of the Ligand/FAP-II complex during the MD simulation at 100, 200, 300, 400, and 500 ns

For clarity, the water molecules have been removed. The ligand is plotted using stick style, while human FAP is presented in cartoon style.

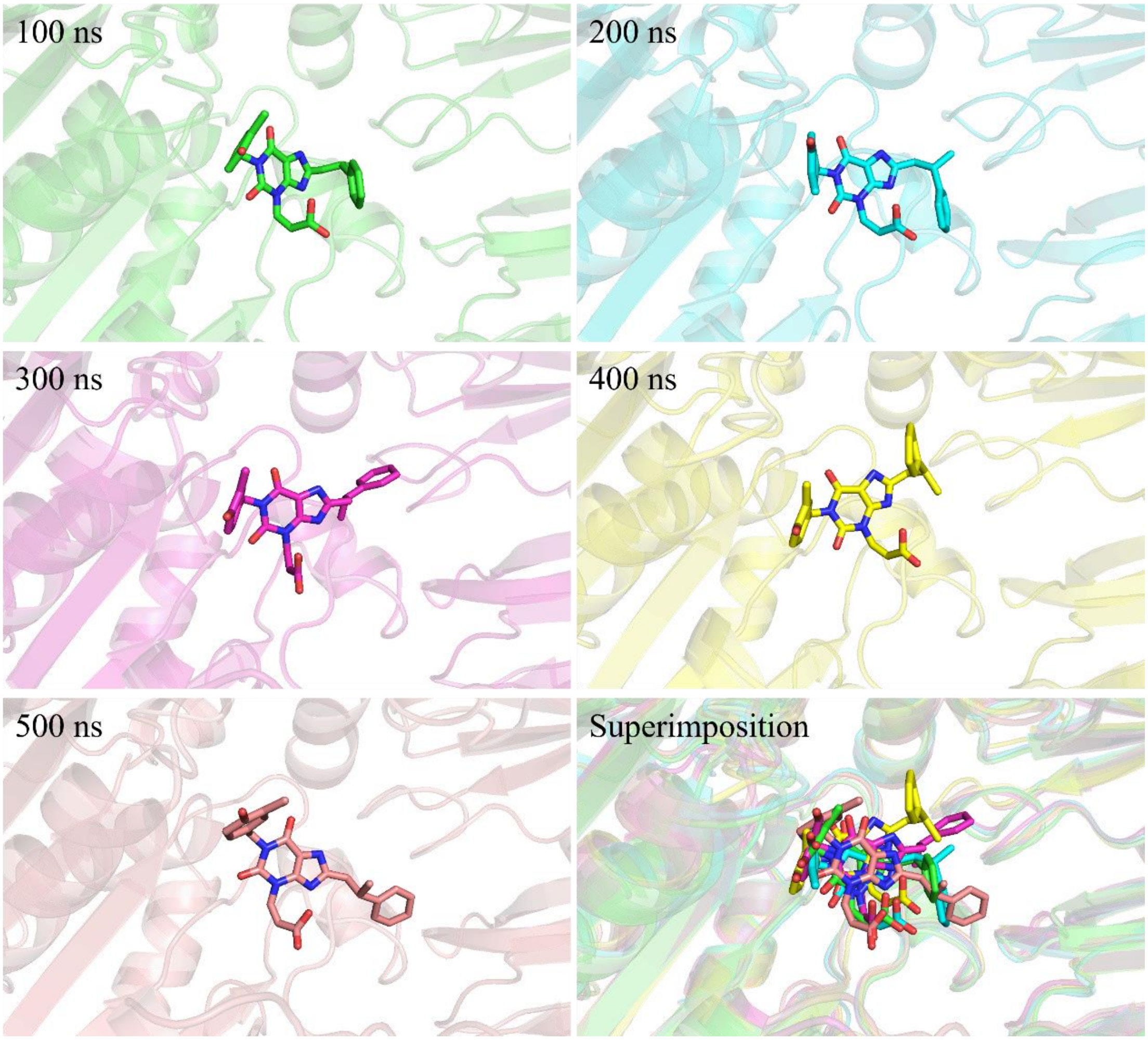

Figure S21 Snapshots of the Ligand/FAP-III complex during the MD simulation at 100, 200, 300, 400, and 500 ns

For clarity, the water molecules have been removed. The ligand is plotted using stick style, while human FAP is presented in cartoon style.

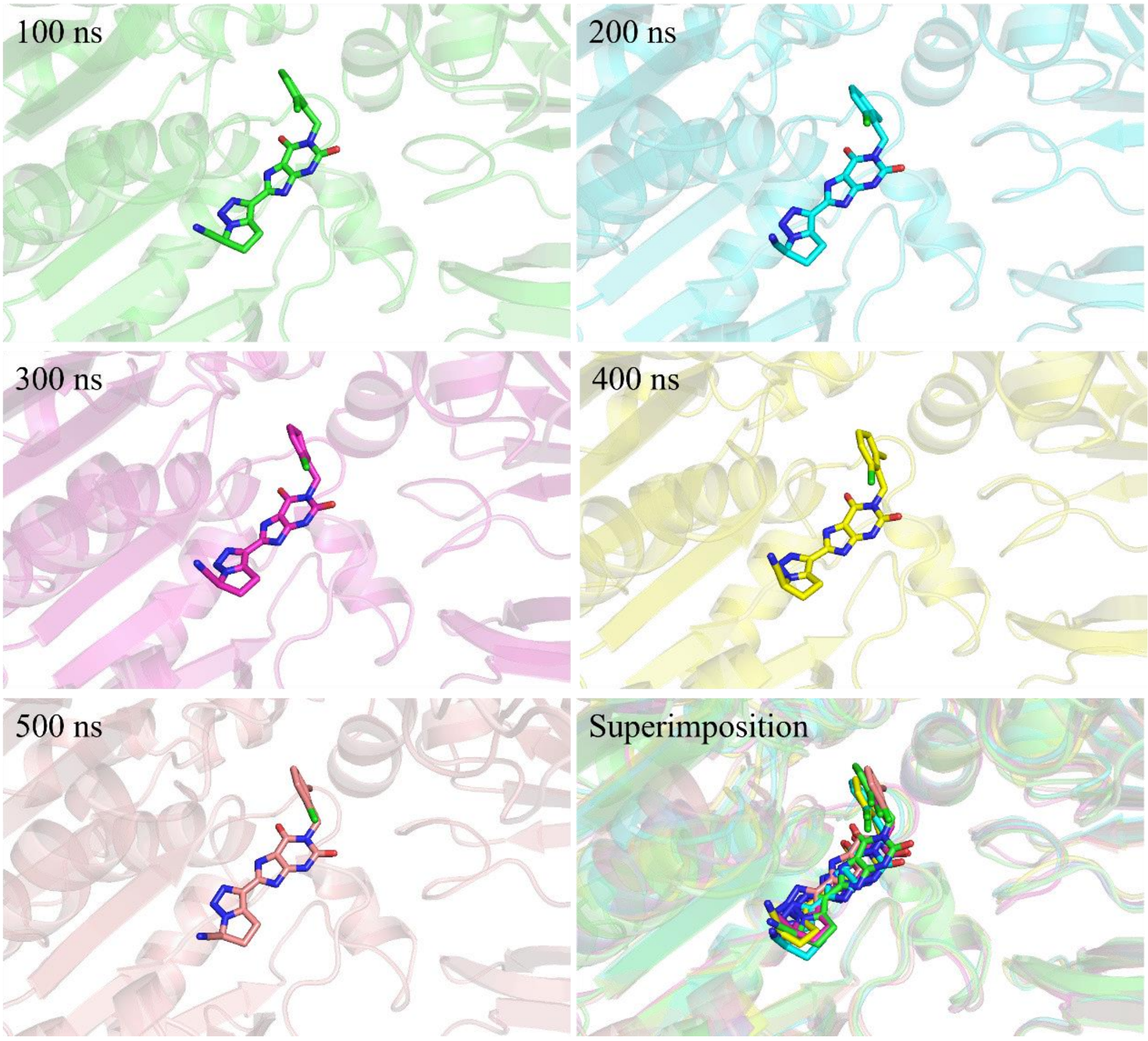

Figure S22 Snapshots of the Ligand/FAP-IV complex during the MD simulation at 100, 200, 300, 400, and 500 ns

For clarity, the water molecules have been removed. The ligand is plotted using stick style, while human FAP is presented in cartoon style.

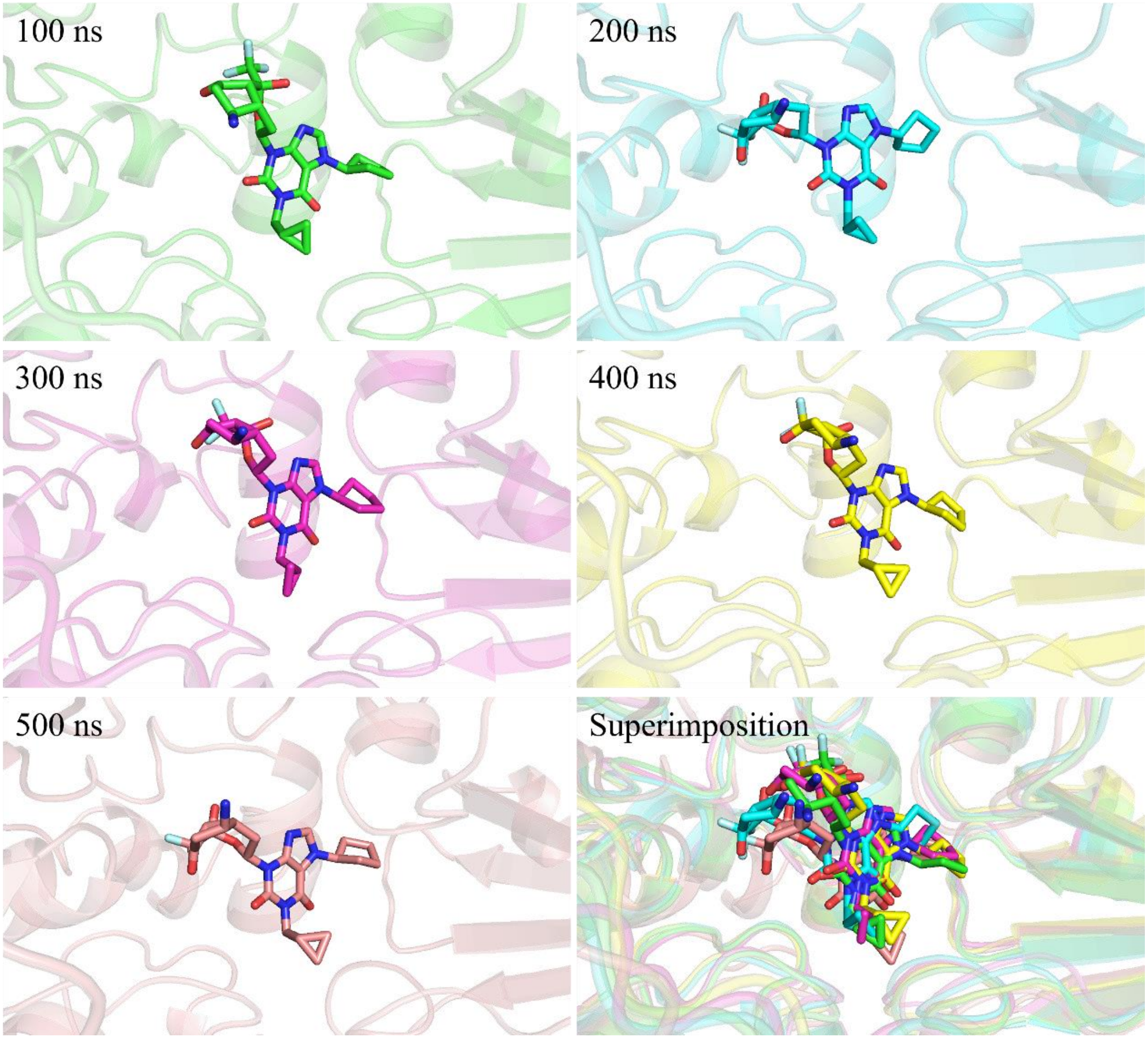

Figure S23 Snapshots of the Ligand/FAP-V complex during the MD simulation at 100, 200, 300, 400, and 500 ns

For clarity, the water molecules have been removed. The ligand is plotted using stick style, while human FAP is presented in cartoon style.

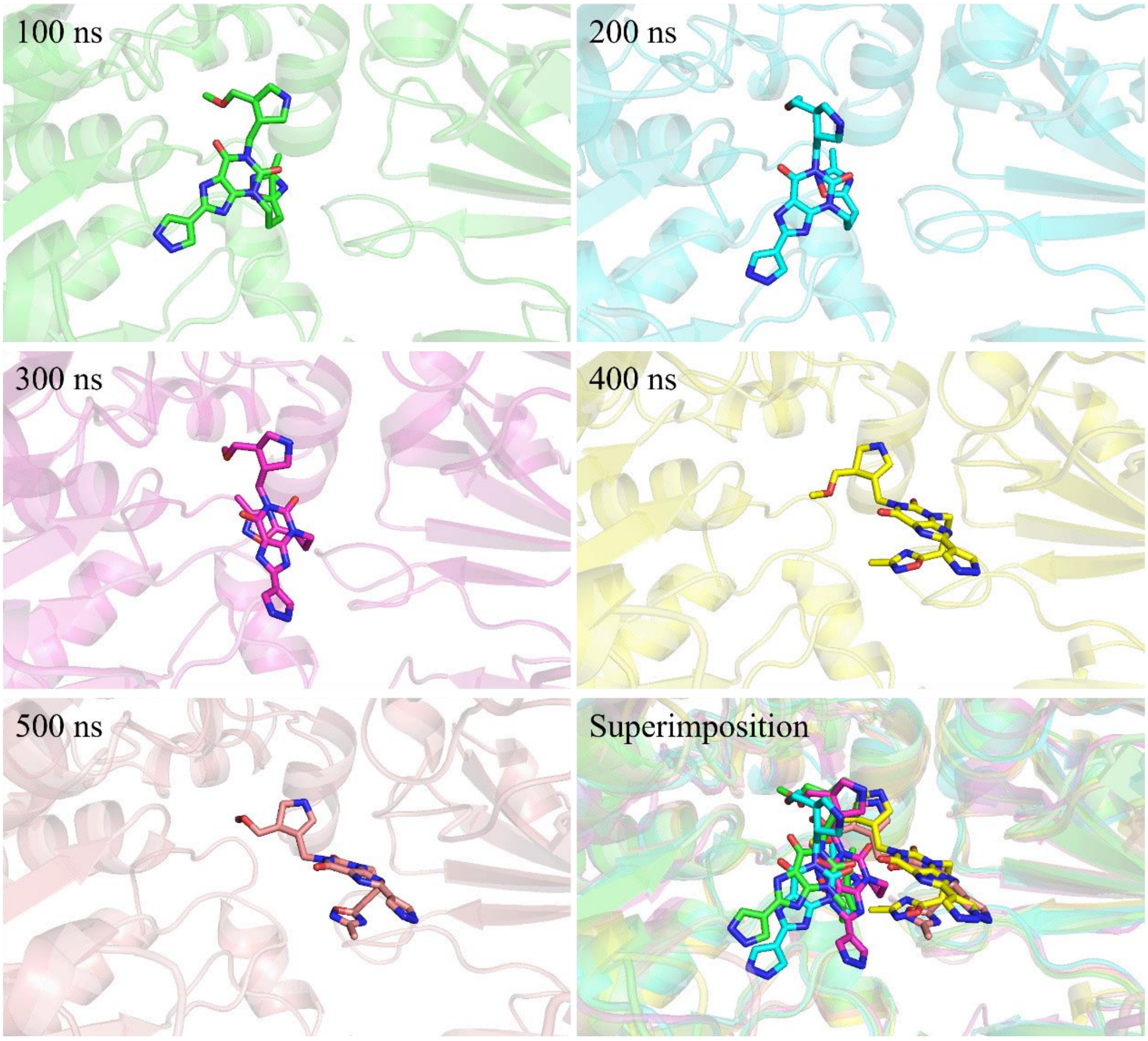

Figure S24 Snapshots of the Ligand/FAP-VI complex during the MD simulation at 100, 200, 300, 400, and 500 ns

For clarity, the water molecules have been removed. The ligand is plotted using stick style, while human FAP is presented in cartoon style.

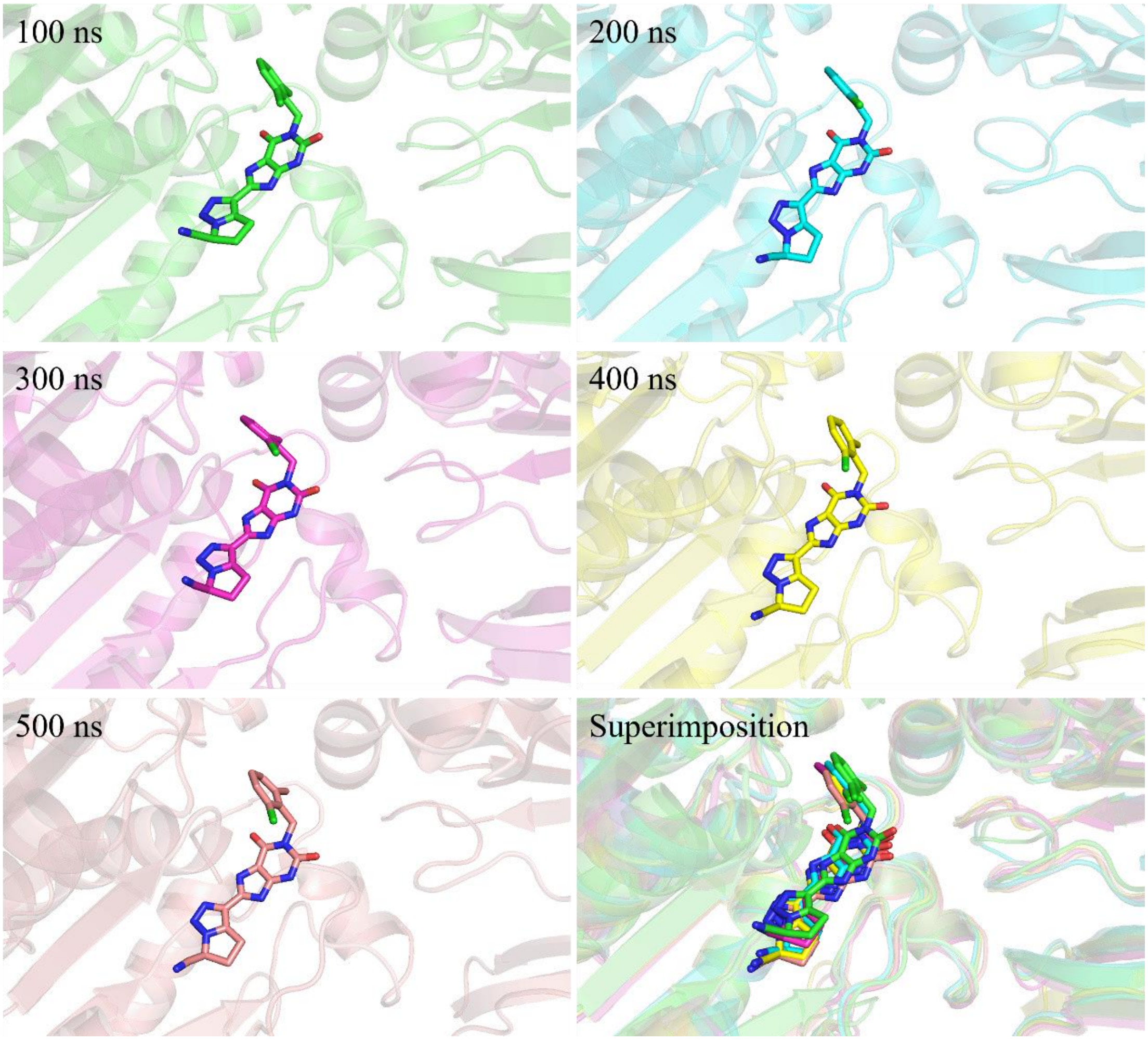

Figure S25 Snapshots of the Ligand/FAP-VII complex during the MD simulation at 100, 200, 300, 400, and 500 ns

For clarity, the water molecules have been removed. The ligand is plotted using stick style, while human FAP is presented in cartoon style.

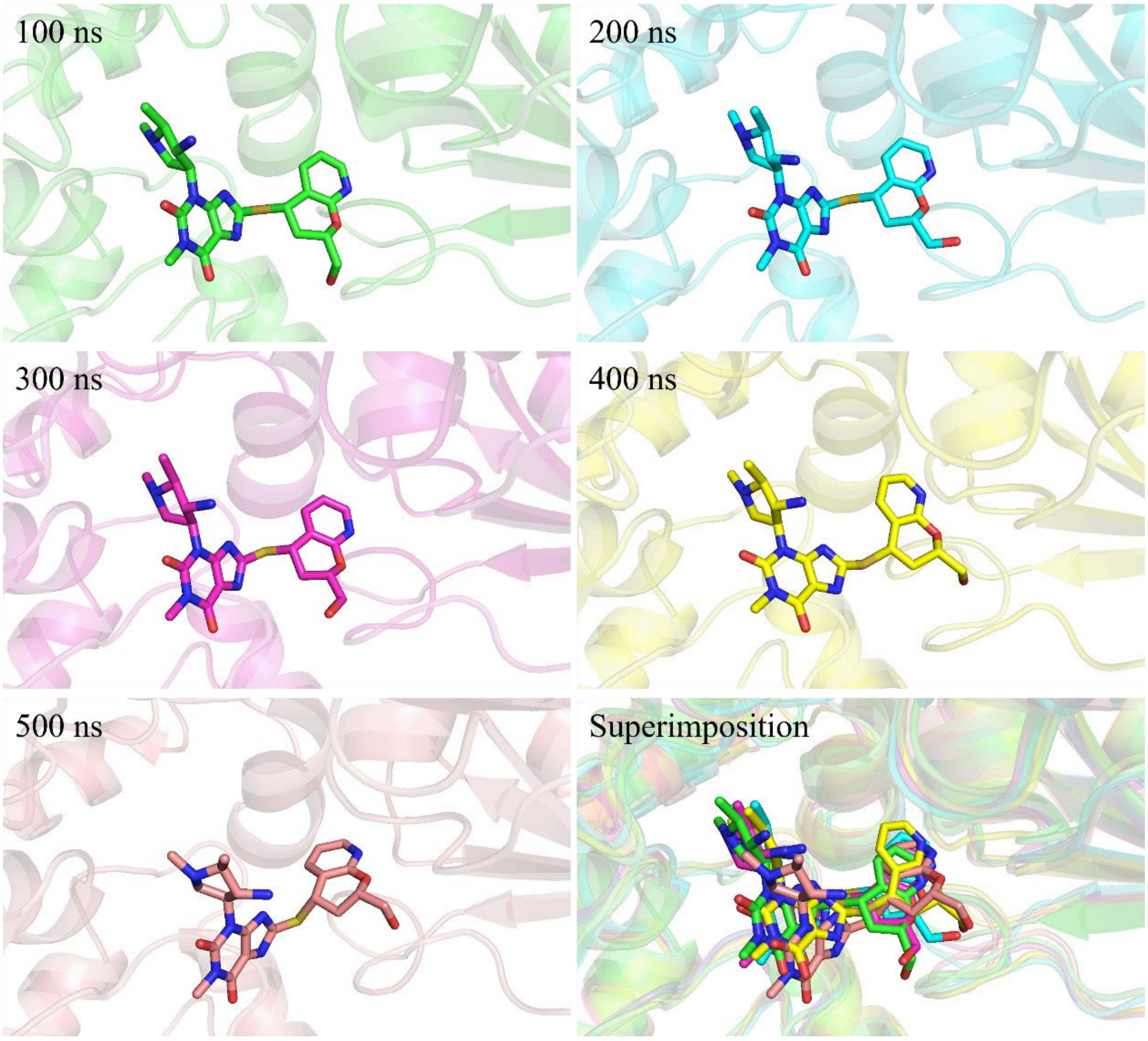

Figure S26 Snapshots of the Ligand/FAP-VIII complex during the MD simulation at 100, 200, 300, 400, and 500 ns

For clarity, the water molecules have been removed. The ligand is plotted using stick style, while human FAP is presented in cartoon style.

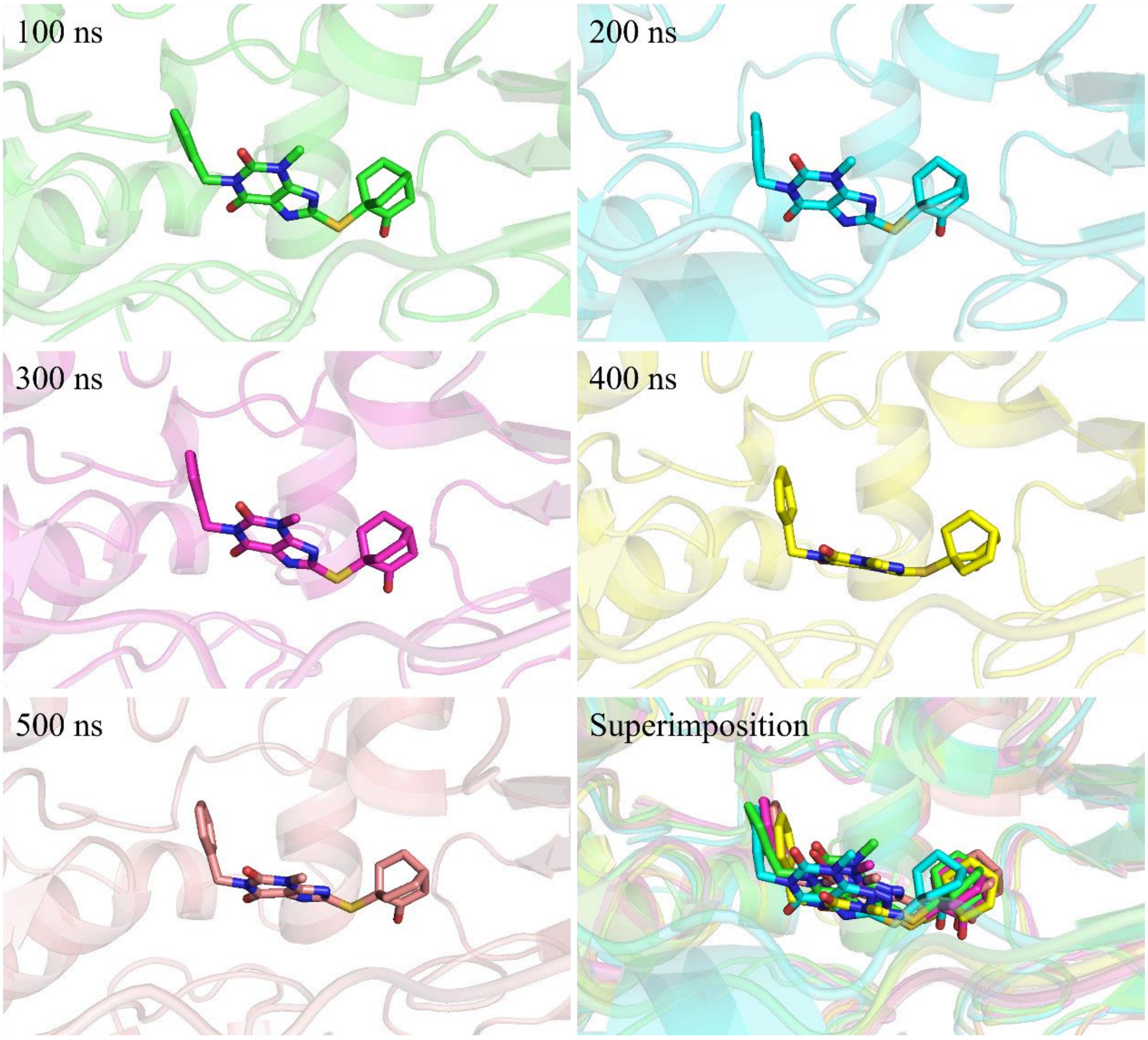

Figure S27 Snapshots of the Ligand/FAP-IX complex during the MD simulation at 100, 200, 300, 400, and 500 ns

For clarity, the water molecules have been removed. The ligand is plotted using stick style, while human FAP is presented in cartoon style.

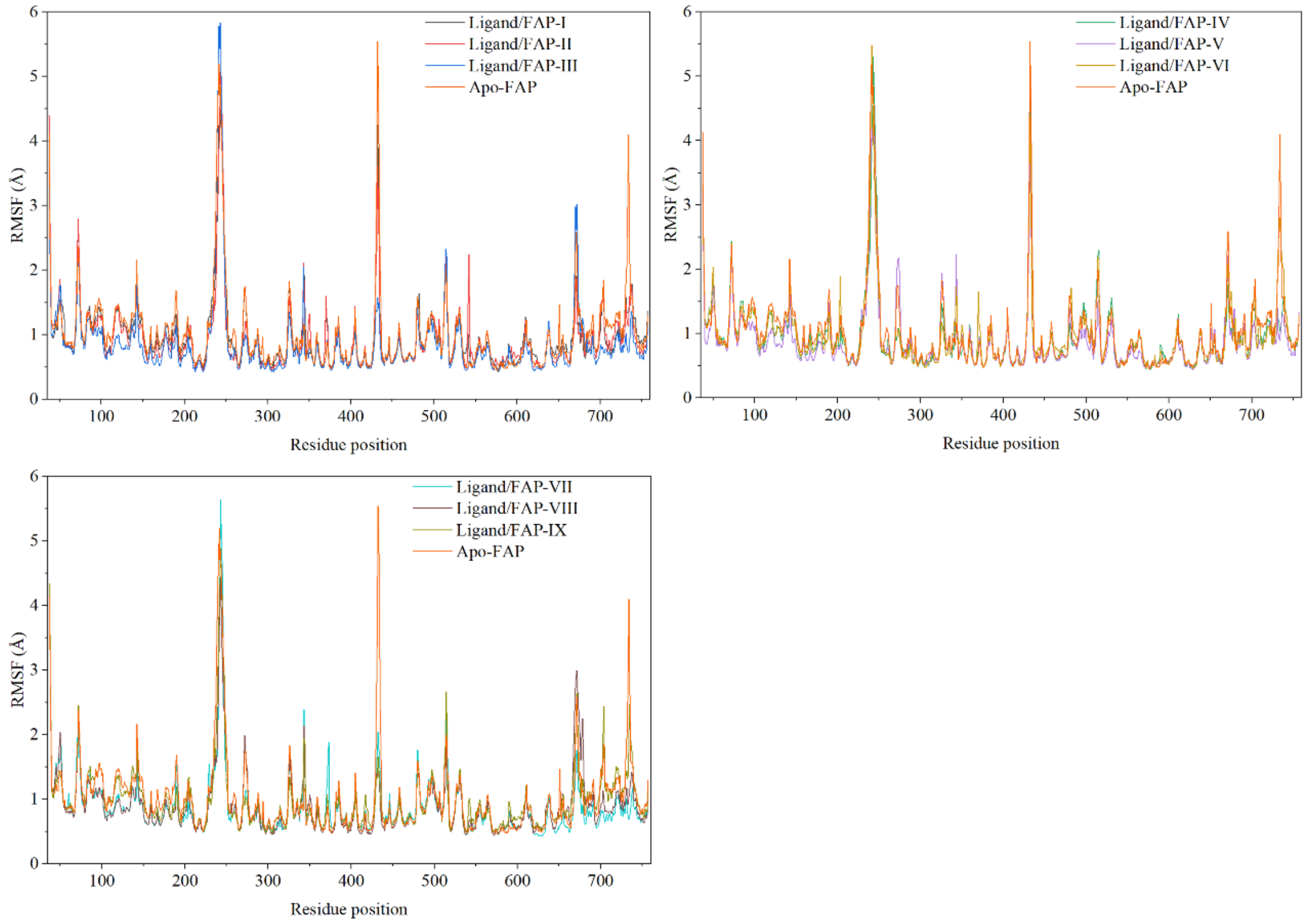

Figure S28 RMSF variations for Cα atom of human FAP for ligand/FAP and Apo-FAP systems from the 500 ns MD simulation

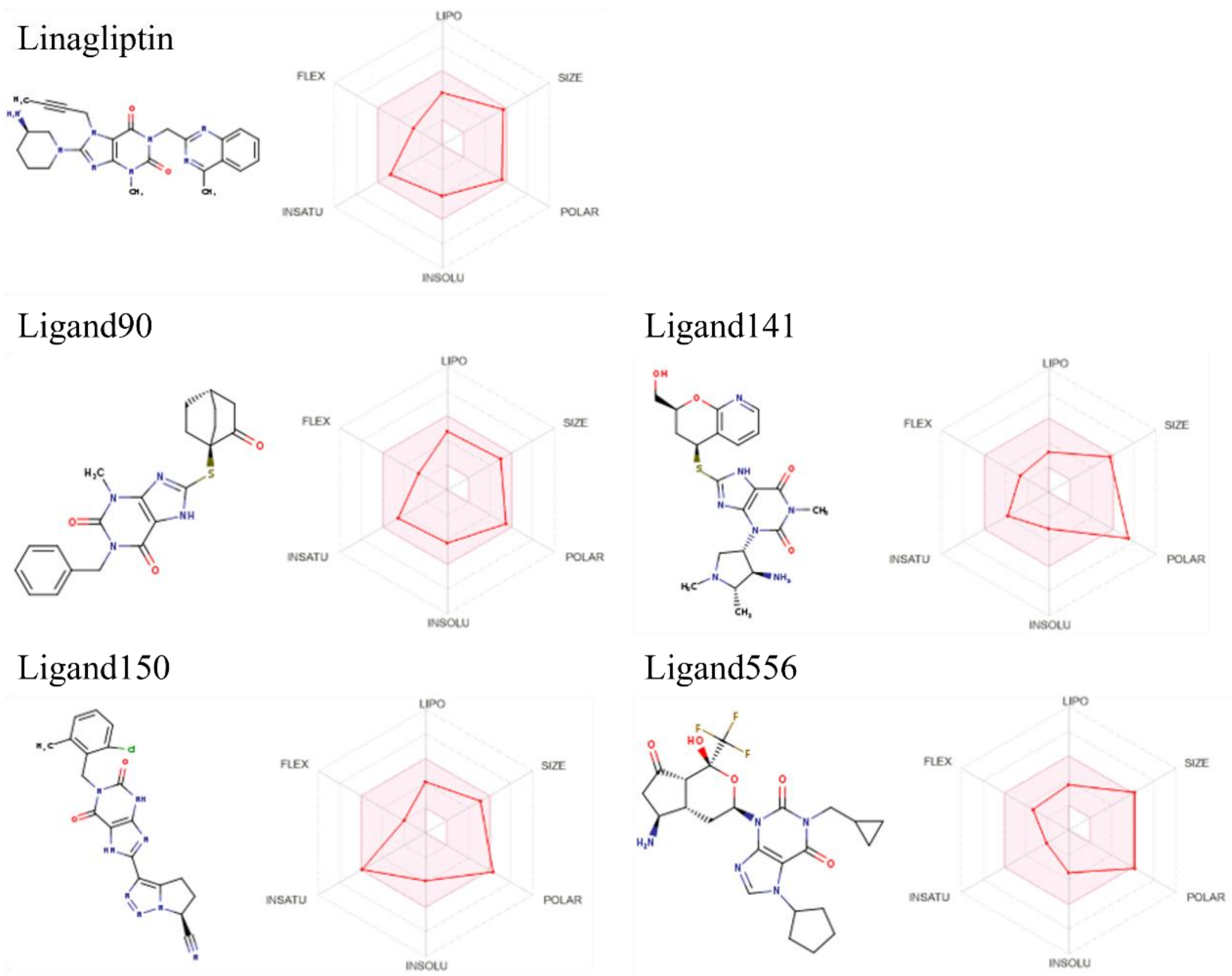

Figure S29 Predicted ADME properties and drug-likeness profiles for the top-ranked linagliptin analogs

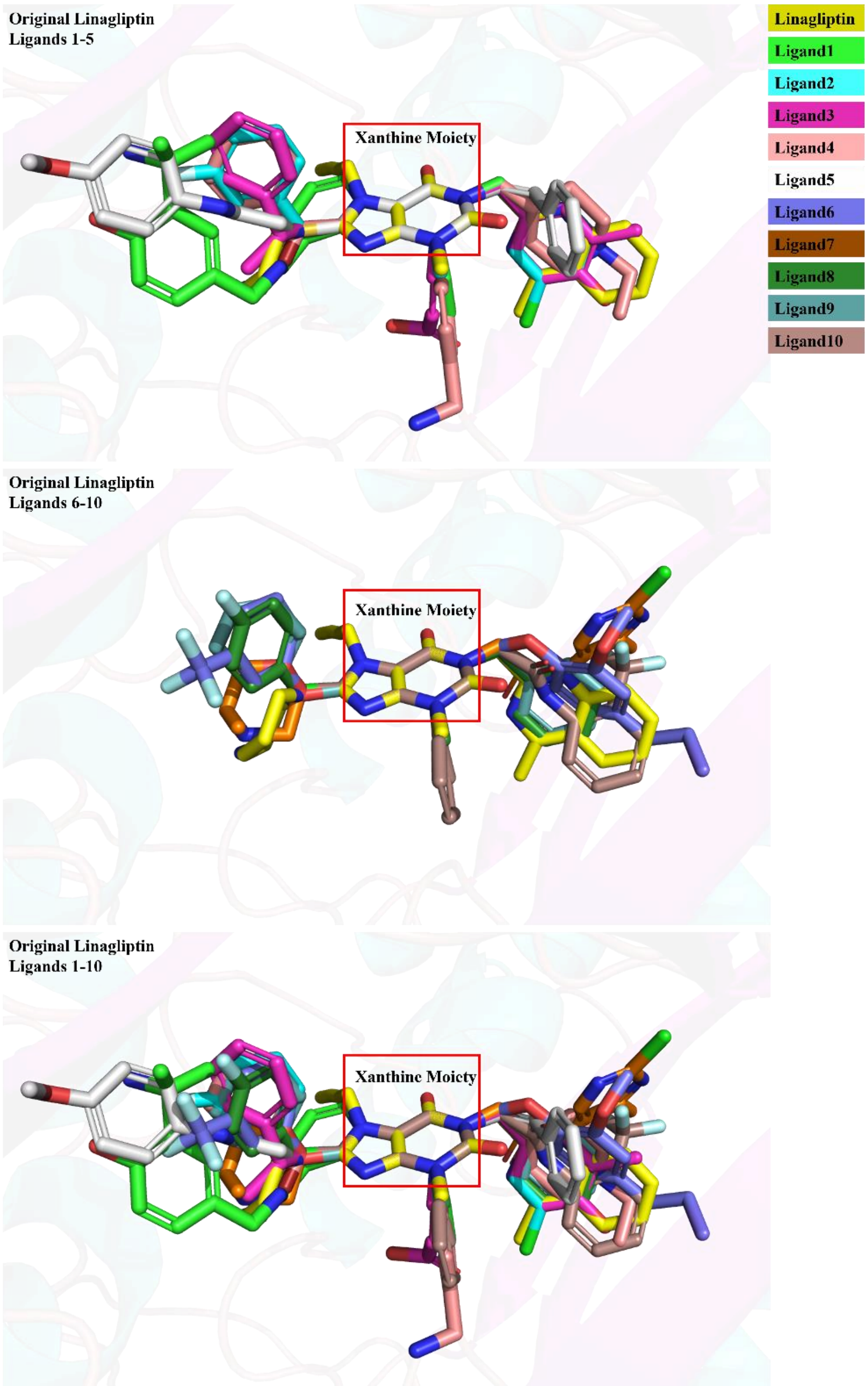

Figure S30 Preservation of the R2 (xanthine) scaffold in AI-generated ligands

Comparison of the binding poses of linagliptin and ten representative AI-generated candidates (Ligands 1–10) within the FAP active site. Superimposed view emphasizing the conserved orientation of the R2/xanthine moiety across all ligands. The R2 group (xanthine core) was fixed during generation, while R1, R3, and R4 substituents were modified.

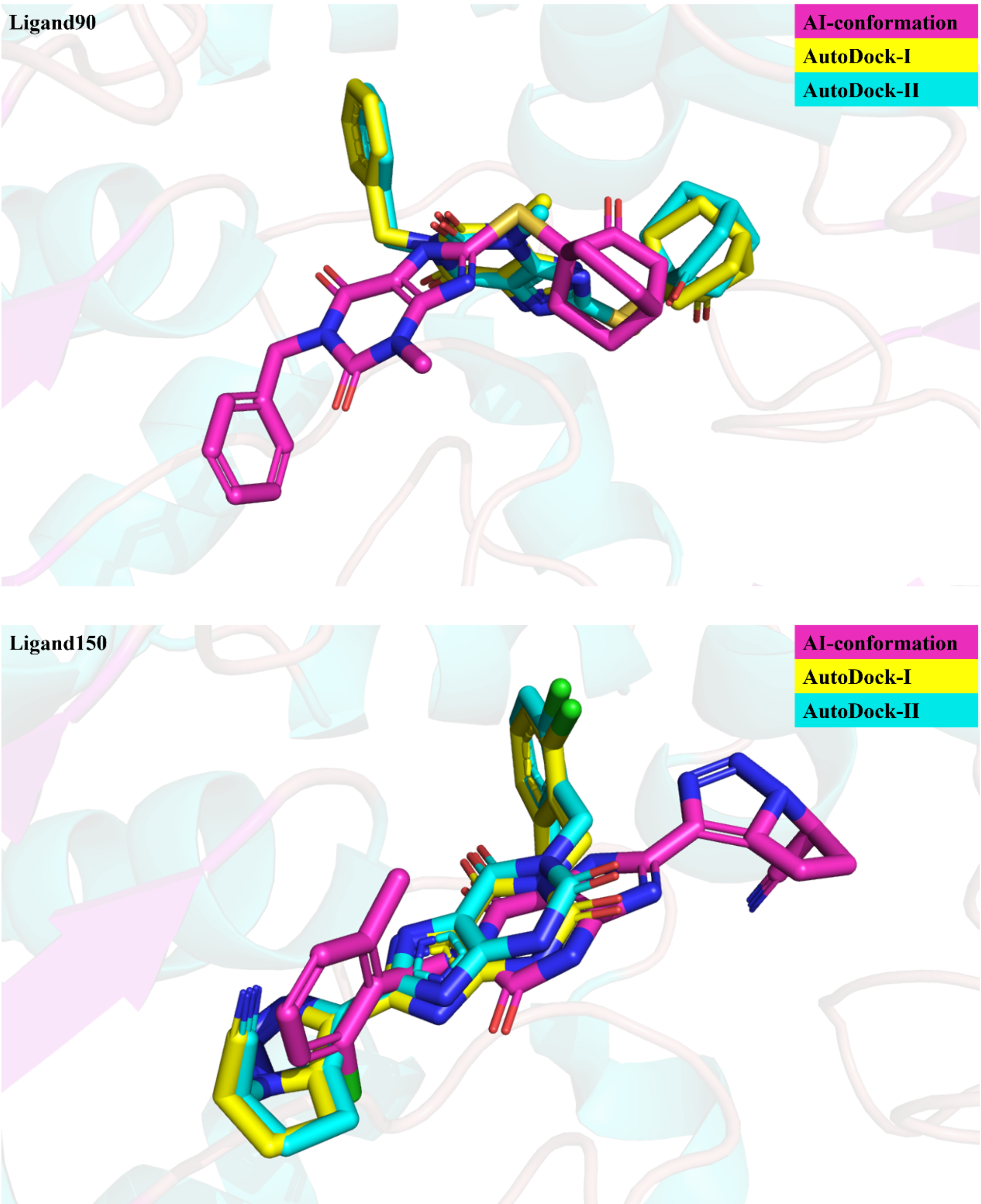

Figure S31 Aligned conformations of Ligand90 and Ligand150 using AI and molecular docking

Table S1 Score value for linagliptin analogues

Label	DeepLigBuilder		AutoDock-I		AutoDock-II	
	Minimized Affinity	Minimized RMSD	Lowest Binding Energy (kcal/mol)	Mean Binding Energy (kcal/mol)	Lowest Binding Energy (kcal/mol)	Mean Binding Energy (kcal/mol)
Ligand1	-7.01	1.00	-8.17	-7.53	-9.52	-8.49
Ligand2	-6.96	1.91	-7.95	-7.68	-8.00	-7.68
Ligand3	-6.51	2.98	-7.88	-7.31	-8.15	-7.45
Ligand4	-6.48	2.30	-8.77	-7.81	-8.15	-7.63
Ligand5	-6.44	3.09	-7.27	-7.27	-8.68	-8.03
Ligand6	-6.40	2.70	-7.31	-6.53	-7.33	-6.57
Ligand7	-6.35	2.07	-8.12	-8.12	-8.46	-8.24
Ligand8	-6.27	0.31	-7.59	-7.21	-7.82	-7.24
Ligand9	-6.22	1.57	-7.62	-7.29	-7.65	-7.37
Ligand10	-6.13	3.65	-7.82	-7.02	-7.90	-7.72
Ligand33	-5.61	2.47	-8.50	-7.93	-9.59	-9.37
Ligand47	-5.46	3.94	-9.41	-9.41	-9.33	-8.61
Ligand82	-5.13	1.35	-8.15	-7.07	-9.60	-8.88
Ligand90	-5.09	1.38	-8.77	-8.77	-10.06	-9.14
Ligand94	-5.06	0.59	-9.22	-8.80	-9.13	-8.70
Ligand141	-4.88	0.96	-9.21	-8.07	-10.09	-8.52
Ligand150	-4.83	2.23	-9.87	-9.18	-10.31	-9.53
Ligand188	-4.72	1.49	-8.37	-7.94	-9.74	-9.66
Ligand258	-4.53	2.75	-9.47	-9.40	-9.43	-9.35
Ligand285	-4.47	5.44	-9.37	-8.15	-8.89	-7.63
Ligand402	-4.18	1.86	-9.52	-8.64	-9.65	-8.73
Ligand427	-4.11	2.32	-9.75	-8.36	-9.70	-8.59
Ligand468	-4.02	1.43	-9.29	-8.63	-9.58	-8.38
Ligand556	-3.82	2.17	-9.81	-8.87	-8.85	-8.85
Ligand807	-3.34	3.26	-9.57	-8.68	-9.67	-9.22

The full information for Table S1 can be obtained from Table S12.

Table S2 RMSD value for the ligand/FAP complex systems during 500-ns MD simulations

RMSD (Å)	FAP	Ligand
Ligand/FAP-I	1.67 ± 0.20*	8.86 ± 3.79
Ligand/FAP-II	1.62 ± 0.20	6.26 ± 1.89
Ligand/FAP-III	1.45 ± 0.24	5.03 ± 0.61
Ligand/FAP-IV	1.52 ± 0.17	2.06 ± 0.31
Ligand/FAP-V	1.46 ± 0.18	3.37 ± 0.49
Ligand/FAP-VI	1.55 ± 0.25	5.37 ± 2.58
Ligand/FAP-VII	1.45 ± 0.16	1.78 ± 0.23
Ligand/FAP-VIII	1.57 ± 0.15	2.90 ± 0.42
Ligand/FAP-IX	1.36 ± 0.23	1.70 ± 0.36

*Data are represented as mean ± standard deviation.

Table S3 Binding energies (ΔE_{bind}^{cal}) for Ligand/FAP-I complex system

Energy (kcal/mol)	Complex	Receptor	Ligand	Delta
E_{vdW}	-6123.14 ± 35.28	-6089.44 ± 35.46	-1.08 ± 2.03	-32.62 ± 4.46
E_{ele}	-51441.59 ± 169.76	-51590.65 ± 170.43	163.95 ± 2.49	-14.89 ± 5.91
E_{polar}	-7668.13 ± 140.43	-7669.15 ± 141.40	-31.25 ± 1.09	32.26 ± 5.89
$E_{nonpolar}$	217.57 ± 3.08	217.91 ± 3.16	4.07 ± 0.13	-4.41 ± 0.64
E_{gas}	-12594.01 ± 176.74	-12292.41 ± 177.94	-254.10 ± 5.72	-47.50 ± 7.88
E_{solv}	-7450.57 ± 138.68	-7451.24 ± 139.58	-27.18 ± 1.04	27.86 ± 5.49
ΔE_{bind}^{cal}	-20044.58 ± 81.33	-19743.65 ± 81.15	-281.28 ± 5.58	-19.64 ± 3.53

The binding energies (ΔE_{bind}^{cal}) for ligand/FAP complex and decomposition to electrostatic interaction (E_{ele}), van der Waals interaction (E_{vdW}), polar solvation free energies (E_{polar}), and nonpolar solvation free energies ($E_{nonpolar}$). Energy values are presented in kcal/mol. Uncertainties were calculated as the root mean square error for all frames extracted from the trajectories.

Table S4 Binding energies (ΔE_{bind}^{cal}) for Ligand/FAP-II complex system

Energy (kcal/mol)	Complex	Receptor	Ligand	Delta
E_{vdW}	-6112.32 ± 34.16	-6071.35 ± 33.85	-4.19 ± 0.76	-36.78 ± 2.42
E_{ele}	-51511.92 ± 167.68	-51616.53 ± 168.03	114.80 ± 2.07	-10.19 ± 3.56
E_{polar}	-7687.95 ± 140.91	-7693.03 ± 141.43	-23.00 ± 0.63	28.08 ± 3.00
$E_{nonpolar}$	218.81 ± 2.90	220.04 ± 2.88	3.48 ± 0.02	-4.70 ± 0.20
E_{gas}	-12498.11 ± 171.92	-12268.11 ± 171.94	-183.04 ± 4.53	-46.96 ± 3.69
E_{solv}	-7469.14 ± 139.42	-7472.99 ± 139.92	-19.52 ± 0.63	23.37 ± 2.93
ΔE_{bind}^{cal}	-19967.25 ± 79.37	-19741.10 ± 79.18	-202.56 ± 4.48	-23.59 ± 2.89

The binding energies (ΔE_{bind}^{cal}) for ligand/FAP complex and decomposition to electrostatic interaction (E_{ele}), van der Waals interaction (E_{vdW}), polar solvation free energies (E_{polar}), and nonpolar solvation free energies ($E_{nonpolar}$). Energy values are presented in kcal/mol. Uncertainties were calculated as the root mean square error for all frames extracted from the trajectories.

Table S5 Binding energies (ΔE_{bind}^{cal}) for Ligand/FAP-III complex system

Energy (kcal/mol)	Complex	Receptor	Ligand	Delta
E_{vdW}	-6126.12 ± 36.64	-6082.97 ± 36.55	-7.33 ± 1.67	-35.82 ± 2.99
E_{ele}	-51367.57 ± 148.52	-51372.46 ± 148.30	23.12 ± 3.67	-18.23 ± 6.77
E_{polar}	-7874.94 ± 124.48	-7878.01 ± 123.82	-35.82 ± 1.49	38.89 ± 6.22
$E_{nonpolar}$	221.00 ± 3.28	220.44 ± 3.36	4.14 ± 0.10	-3.57 ± 0.39
E_{gas}	-12241.03 ± 155.46	-12059.72 ± 155.10	-127.26 ± 5.69	-54.06 ± 7.94
E_{solv}	-7653.94 ± 122.83	-7657.58 ± 122.13	-31.68 ± 1.46	35.31 ± 6.05
ΔE_{bind}^{cal}	-19894.97 ± 80.92	-19717.29 ± 80.78	-158.93 ± 5.41	-18.74 ± 3.14

The binding energies (ΔE_{bind}^{cal}) for ligand/FAP complex and decomposition to electrostatic interaction (E_{ele}), van der Waals interaction (E_{vdW}), polar solvation free energies (E_{polar}), and nonpolar solvation free energies ($E_{nonpolar}$). Energy values are presented in kcal/mol. Uncertainties were calculated as the root mean square error for all frames extracted from the trajectories.

Table S6 Binding energies (ΔE_{bind}^{cal}) for Ligand/FAP-IV complex system

Energy (kcal/mol)	Complex	Receptor	Ligand	Delta
E_{vdW}	-6151.25 ± 34.20	-6099.57 ± 34.14	-4.52 ± 1.12	-47.16 ± 2.41
E_{ele}	-51627.38 ± 163.00	-51630.75 ± 162.84	10.83 ± 1.57	-7.45 ± 3.76
E_{polar}	-7688.33 ± 143.91	-7681.68 ± 143.60	-34.69 ± 0.61	28.04 ± 2.95
$E_{nonpolar}$	216.75 ± 3.08	218.35 ± 3.08	3.37 ± 0.02	-4.96 ± 0.16
E_{gas}	-12405.52 ± 183.31	-12264.76 ± 183.00	-86.15 ± 4.90	-54.61 ± 3.97
E_{solv}	-7471.57 ± 142.04	-7463.33 ± 141.72	-31.32 ± 0.61	23.08 ± 2.93
ΔE_{bind}^{cal}	-19877.09 ± 82.45	-19728.09 ± 82.16	-117.47 ± 4.88	-31.53 ± 2.72

The binding energies (ΔE_{bind}^{cal}) for ligand/FAP complex and decomposition to electrostatic interaction (E_{ele}), van der Waals interaction (E_{vdW}), polar solvation free energies (E_{polar}), and nonpolar solvation free energies ($E_{nonpolar}$). Energy values are presented in kcal/mol. Uncertainties were calculated as the root mean square error for all frames extracted from the trajectories.

Table S7 Binding energies (ΔE_{bind}^{cal}) for Ligand/FAP-V complex system

Energy (kcal/mol)	Complex	Receptor	Ligand	Delta
E_{vdW}	-6110.36 ± 32.97	-6054.74 ± 32.79	-5.89 ± 1.55	-49.73 ± 2.52
E_{ele}	-51171.75 ± 161.00	-51267.84 ± 161.10	117.49 ± 4.56	-21.41 ± 7.84
E_{polar}	-7898.49 ± 123.53	-7917.00 ± 123.31	-31.89 ± 1.89	50.41 ± 5.57
$E_{nonpolar}$	224.13 ± 2.55	226.19 ± 2.57	4.26 ± 0.03	-6.32 ± 0.33
E_{gas}	-12050.23 ± 154.72	-11959.02 ± 154.49	-20.08 ± 6.12	-71.13 ± 8.37
E_{solv}	-7674.36 ± 122.23	-7690.81 ± 122.01	-27.63 ± 1.88	44.08 ± 5.47
ΔE_{bind}^{cal}	-19724.59 ± 78.45	-19649.83 ± 78.19	-47.71 ± 5.66	-27.04 ± 5.82

The binding energies (ΔE_{bind}^{cal}) for ligand/FAP complex and decomposition to electrostatic interaction (E_{ele}), van der Waals interaction (E_{vdW}), polar solvation free energies (E_{polar}), and nonpolar solvation free energies ($E_{nonpolar}$). Energy values are presented in kcal/mol. Uncertainties were calculated as the root mean square error for all frames extracted from the trajectories.

Table S8 Binding energies (ΔE_{bind}^{cal}) for Ligand/FAP-VI complex system

Energy (kcal/mol)	Complex	Receptor	Ligand	Delta
E_{vdW}	-6128.99 ± 35.52	-6076.29 ± 35.25	-10.66 ± 1.30	-42.03 ± 4.08
E_{ele}	-51424.02 ± 149.71	-51428.84 ± 149.97	16.75 ± 2.10	-11.93 ± 4.82
E_{polar}	-7839.16 ± 119.56	-7839.60 ± 119.76	-40.96 ± 1.17	41.40 ± 4.84
$E_{nonpolar}$	220.92 ± 2.61	222.13 ± 2.55	4.10 ± 0.04	-5.32 ± 0.42
E_{gas}	-12198.45 ± 150.41	-12074.35 ± 150.37	-70.14 ± 5.77	-53.96 ± 6.24
E_{solv}	-7618.25 ± 118.37	-7617.47 ± 118.56	-36.86 ± 1.17	36.09 ± 4.69
ΔE_{bind}^{cal}	-19816.69 ± 82.93	-19691.82 ± 82.51	-107.00 ± 5.64	-17.87 ± 3.28

The binding energies (ΔE_{bind}^{cal}) for ligand/FAP complex and decomposition to electrostatic interaction (E_{ele}), van der Waals interaction (E_{vdW}), polar solvation free energies (E_{polar}), and nonpolar solvation free energies ($E_{nonpolar}$). Energy values are presented in kcal/mol. Uncertainties were calculated as the root mean square error for all frames extracted from the trajectories.

Table S9 Binding energies (ΔE_{bind}^{cal}) for Ligand/FAP-VII complex system

Energy (kcal/mol)	Complex	Receptor	Ligand	Delta
E_{vdW}	-6128.27 ± 34.72	-6082.28 ± 34.53	-4.57 ± 1.06	-41.42 ± 2.20
E_{ele}	-51395.72 ± 165.65	-51400.03 ± 165.30	10.27 ± 1.49	-5.96 ± 3.45
E_{polar}	-7738.31 ± 121.16	-7726.85 ± 121.04	-31.72 ± 0.63	20.26 ± 2.51
$E_{nonpolar}$	220.26 ± 2.52	221.30 ± 2.51	3.37 ± 0.02	-4.42 ± 0.13
E_{gas}	-12215.48 ± 162.06	-12080.26 ± 161.82	-87.84 ± 4.59	-47.37 ± 3.51
E_{solv}	-7518.05 ± 120.27	-7505.55 ± 120.12	-28.34 ± 0.62	15.84 ± 2.51
ΔE_{bind}^{cal}	-19733.53 ± 85.88	-19585.81 ± 85.66	-116.18 ± 4.56	-31.54 ± 2.25

The binding energies (ΔE_{bind}^{cal}) for ligand/FAP complex and decomposition to electrostatic interaction (E_{ele}), van der Waals interaction (E_{vdW}), polar solvation free energies (E_{polar}), and nonpolar solvation free energies ($E_{nonpolar}$). Energy values are presented in kcal/mol. Uncertainties were calculated as the root mean square error for all frames extracted from the trajectories.

Table S10 Binding energies (ΔE_{bind}^{cal}) for Ligand/FAP-VIII complex system

Energy (kcal/mol)	Complex	Receptor	Ligand	Delta
E_{vdW}	-6135.36 ± 40.40	-6084.47 ± 40.73	-3.76 ± 1.51	-47.13 ± 3.75
E_{ele}	-51497.20 ± 182.08	-51365.02 ± 187.46	-87.79 ± 8.11	-44.40 ± 10.28
E_{polar}	-7807.12 ± 137.26	-7837.68 ± 138.79	-38.72 ± 1.81	69.28 ± 7.13
$E_{nonpolar}$	218.22 ± 3.78	220.19 ± 3.78	4.27 ± 0.03	-6.25 ± 0.28
E_{gas}	-12282.31 ± 166.26	-12069.55 ± 168.89	-121.23 ± 6.12	-91.53 ± 8.73
E_{solv}	-7588.90 ± 136.59	-7617.48 ± 138.23	-34.45 ± 1.8	63.03 ± 7.14
ΔE_{bind}^{cal}	-19871.21 ± 80.12	-19687.03 ± 80.22	-155.68 ± 5.55	-28.50 ± 4.21

The binding energies (ΔE_{bind}^{cal}) for ligand/FAP complex and decomposition to electrostatic interaction (E_{ele}), van der Waals interaction (E_{vdW}), polar solvation free energies (E_{polar}), and nonpolar solvation free energies ($E_{nonpolar}$). Energy values are presented in kcal/mol. Uncertainties were calculated as the root mean square error for all frames extracted from the trajectories.

Table S11 Binding energies (ΔE_{bind}^{cal}) for Ligand/FAP-IX complex system

Energy (kcal/mol)	Complex	Receptor	Ligand	Delta
E_{vdW}	-6096.81 ± 33.72	-6050.43 ± 33.63	-4.97 ± 0.96	-41.42 ± 2.36
E_{ele}	-51642.13 ± 173.19	-51570.42 ± 172.33	-53.72 ± 1.91	-18 ± 3.67
E_{polar}	-7691.48 ± 138.27	-7706.1 ± 137.72	-23.36 ± 0.57	37.98 ± 3.46
$E_{nonpolar}$	222.23 ± 3.03	223.52 ± 3.09	3.63 ± 0.03	-4.92 ± 0.23
E_{gas}	-12313.33 ± 174.9	-12218.89 ± 174.3	-35.02 ± 4.84	-59.41 ± 3.72
E_{solv}	-7469.25 ± 136.29	-7482.58 ± 135.68	-19.73 ± 0.56	33.06 ± 3.46
ΔE_{bind}^{cal}	-19782.57 ± 82.65	-19701.48 ± 82.5	-54.75 ± 4.83	-26.35 ± 2.69

The binding energies (ΔE_{bind}^{cal}) for ligand/FAP complex and decomposition to electrostatic interaction (E_{ele}), van der Waals interaction (E_{vdW}), polar solvation free energies (E_{polar}), and nonpolar solvation free energies ($E_{nonpolar}$). Energy values are presented in kcal/mol. Uncertainties were calculated as the root mean square error for all frames extracted from the trajectories.
