# Supplementary material for: Designing novel linagliptin analogs through combined generative artificial intelligence, molecular docking, molecular dynamics simulation, and binding energy estimation for targeting fibroblast activation protein
Source: Front Chem. 2026 Sep 9;14:1848970. doi: 10.3389/fchem.2026.1848970 (PMC13597733; doi:10.3389/fchem.2026.1848970)
Supplement: Supplementary file 2 [file DataSheet1.zip › Readme.docx]

File name and description for molecule structures files

| File name | Description |
| --- | --- |
| Linagliptin_FAP.pdb | Initial linagliptin/FAP complex structure |
| Linagliptin_FAP_Redock.pdb | Redock results for linagliptin/FAP complex |
| LigandsFromDeepLigBuilder.sdf | The 1016 molecules generated from DeepLigBuilder |
| LigandsFromDeepLigBuilderAsIndividual | The individual ligand files for 1016 molecules |
| Ligand1_FAP_DeepLigBuilder.pdb | Ligand1/FAP complex structure from DeepLigBuilder |
| Ligand2_FAP_DeepLigBuilder.pdb | Ligand2/FAP complex structure from DeepLigBuilder |
| Ligand3_FAP_DeepLigBuilder.pdb | Ligand3/FAP complex structure from DeepLigBuilder |
| Ligand150_FAP_AutoDock-I.pdb | Ligand150/FAP complex structure from AutoDock-I |
| Ligand427_FAP_AutoDock-I.pdb | Ligand427/FAP complex structure from AutoDock-I |
| Ligand556_FAP_AutoDock-I.pdb | Ligand556/FAP complex structure from AutoDock-I |
| Ligand90_FAP_AutoDock-II.pdb | Ligand90/FAP complex structure from AutoDock-II |
| Ligand141_FAP_AutoDock-II.pdb | Ligand141/FAP complex structure from AutoDock-II |
| Ligand150_FAP_AutoDock-II.pdb | Ligand150/FAP complex structure from AutoDock-II |
| Ligand_FAP_I_MD.pdb | Ligand/FAP-I complex structure from 500th-ns MD |
| Ligand_FAP_II_MD.pdb | Ligand/FAP-II complex structure from 500th-ns MD |
| Ligand_FAP_III_MD.pdb | Ligand/FAP-III complex structure from 500th-ns MD |
| Ligand_FAP_IV_MD.pdb | Ligand/FAP-IV complex structure from 500th-ns MD |
| Ligand_FAP_V_MD.pdb | Ligand/FAP-V complex structure from 500th-ns MD |
| Ligand_FAP_VI_MD.pdb | Ligand/FAP-VI complex structure from 500th-ns MD |
| Ligand_FAP_VII_MD.pdb | Ligand/FAP-VII complex structure from 500-ns MD |
| Ligand_FAP_VIII_MD.pdb | Ligand/FAP-VIII complex structure from 500th-ns MD |
| Ligand_FAP_IX_MD.pdb | Ligand/FAP-IX complex structure from 500th-ns MD |
| TableS12.xlsx | Table S12 Score value for linagliptin analogues |
